# Promises and limitations of current ancient human epigenetic clocks

**DOI:** 10.1016/j.isci.2026.118061

**Published:** 2026-10-28

**Authors:** Youssef Tawfik, Yoan Diekmann, Ludovic Orlando, Joachim Burger, Jens Blöcher

**Affiliations:** 1 Institute of Organismic and Molecular Evolution (iomE), Johannes Gutenberg University of Mainz, 55128 Mainz, Germany; 2 Centre d’Anthropologie et de Génomique de Toulouse, CNRS UMR5288, Université Paul Sabatier, 31000 Toulouse, France

**Keywords:** ancient DNA, DNA methylation, age-at-death, epigenetic clocks, sex estimation, paleodemography

## Abstract

Age-at-death estimation of archaeological human remains is central to paleodemographic research yet remains particularly challenging for adults, where osteological methods often produce imprecise age ranges. Epigenetic clocks can accurately predict chronological age in modern humans, but their applicability to ancient human DNA is unclear due to data limitations and indirect methylation inference. Here, we evaluate existing epigenetic clocks on ancient human methylomes combining high-coverage genomic data and a correction framework. Using DNA methylation estimates inferred across different CpG window sizes, neither direct clock application nor regression-based retraining produced reliable age-at-death estimates, and adult-subadult classification did not return statistically supported age classes. In contrast, X chromosome methylation separated males and females, suggesting that broad biological signals remain detectable at sufficient sequencing depth. These results indicate that current approaches can recover global biological signals but remain insufficiently sensitive to capture gradual age-related variation. Estimating age-at-death from ancient methylomes will therefore require methodological advances beyond correction alone, including reference data and improved models for inferring damage-derived epigenetic signals.

## Highlights

• Sex-specific X chromosome methylation is detectable at sufficient depth
• Ancient methylomes lack the resolution for reliable single-CpG inference
• Correction based on modern data did not help to improve age-at-death estimation
• Bone-specific reference data and models are needed for reliable age estimation

## Graphical abstract

**Figure:**
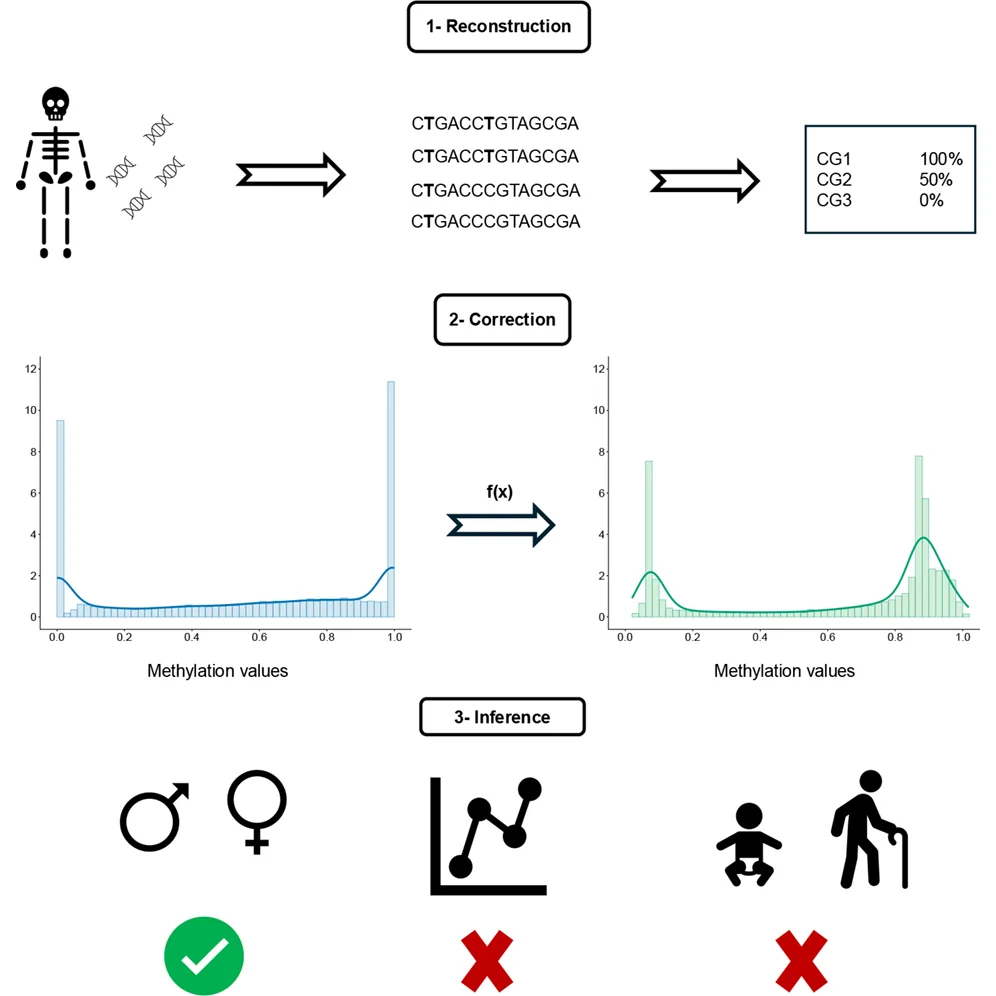

## Teaser

Biological sciences; Genetics; Molecular Genetics

## Introduction

Paleodemography, the study of population dynamics in prehistoric societies, critically depends on accurate age-at-death estimates to reconstruct life-history traits, such as fertility, mortality, and survivorship.^1^^,^^2^ However, traditional osteological methods have inherent limitations, both in modern^3^ and ancient societies.^4^ For subadults, systematic assessment of developmental changes such as tooth eruption and epiphyseal fusion allows for relatively precise age estimates. Once adulthood is reached, skeletal changes become harder to discriminate and correlate only loosely with chronological age due to high variability in the senescence processes.^5^^,^^6^ Furthermore, the “age mimicry” phenomenon, where estimated profiles mirror reference datasets rather than true ages, introduces another layer of systematic bias.^5^^,^^7^^,^^8^ These limitations restrict the resolution of paleodemographic models, ultimately limiting our understanding of how ancient populations responded to changes in lifestyle, environmental shifts, cultural transitions, or demographic pressures.

A potential alternative to osteological age estimation has emerged with the development of epigenetic clocks, which represent one of the most significant methodological advances in aging research. Epigenetic clocks started with the discovery that specific patterns of DNA methylation—a central epigenetic mechanism that regulates gene expression, development, and cellular identity^9^^,^^10^—change predictably with age.^11^ Since then, many epigenetic clocks have been developed across various tissues, based on different modeling approaches.^12^^,^^13^^,^^14^^,^^15^^,^^16^ Recent efforts by Horvath and colleagues have also produced universal pan-tissue clocks by focusing on the age-associated methylation changes that are conserved across mammals.^17^^,^^18^^,^^19^^,^^20^ Epigenetic clocks rely on the direct quantification of DNA methylation and therefore require high-quality genomic DNA with sequencing depth ∼100X per CpG,^21^ often realized through the use of hybridization-based arrays followed by bisulfite or nanopore sequencing.^22^ This robust methodology, well-established in biomedical and forensic contexts,^23^ could offer a promising framework when extended to ancient DNA (aDNA).

Ancient DNA preserves information about DNA methylation states, either through direct chemical retention of methylated CpG dinucleotides or indirectly through postmortem damage patterns that are dependent on original methylation status. Methylated cytosines preferentially deaminate into thymines, while unmethylated cytosines deaminate to uracils,^24^ leaving a molecular signature of past methylation patterns that can be leveraged if specific enzymatic treatments are applied. One such treatment relies on the USER-enzymatic mix, which specifically cleaves uracils, removing postmortem damage signatures on those deaminated methylated CpGs. These sites are sequenced as CpG dinucleotides when not affected by postmortem damage, typically outside the short overhanging ends of DNA templates, or as TpGs otherwise, especially at deaminated methylated CpGs.^24^ This discovery established the foundation of paleoepigenetics and enabled the reconstruction of the first methylomes of ancient hominins,^25^^,^^26^ providing access to regulatory variation not evident from sequencing data of skeletal remains alone.^27^ Several computational frameworks, such as epiPALEOMIX,^28^ DamMet,^29^ and RoAM,^30^ utilize these principles to infer methylation levels in ancient genomes.

While applying epigenetic clocks to ancient DNA holds great potential to help advance paleodemography, and even promises to revive it, direct application has proven to be difficult so far.^25^^,^^28^^,^^31^^,^^32^ However, a recent study on horses introduced a mathematical transformation step adjusting ancient DNA methylation estimates according to modern methylation scales, resulting in improved age-at-death predictions, assuming that CpGs could be covered at high sequencing depth.^33^ Following these results, our aim was to investigate whether similar correction strategies can be extended to ancient human genomes and whether a similar increase in precision can be achieved. We therefore compiled a dataset of high coverage genomes (≥10X) from individuals with reliable anthropological age-at-death estimates to evaluate the applicability of various epigenetic clocks on ancient humans.

## Results and discussion

### Reconstructed ancient methylomes

Our dataset consists of 62 publicly available ancient human genomes (Table S1; Figures 1 and S1) with corresponding anthropological age-at-death estimates (mean age estimates: 1–65 years, 24 subadults and 38 adults, Figure S2) and reported biological sex (24 females and 38 males), each sequenced to high average depth (∼10–34X, see STAR Methods). DNA methylation was reconstructed using DamMet,^29^ which provides probabilistic estimates of the fraction (F) of methylated cytosines at each CpG site. For an accurate assessment of site-specific methylation levels at individual CpGs, substantial sequencing depth is required (≥80X).^29^ To increase precision at lower coverages, CpGs are usually binned within windows of 10–50 CpGs over which estimates are averaged. This approach relies on the strong local correlation of DNA methylation levels among neighboring CpGs that was reported for different tissues.^34^ While this enables estimation of methylation for genomes above 10X, it also leads to a degree of smoothing and a reduction of precision at the single site level.^25^^,^^26^^,^^27^^,^^28^^,^^29^^,^^31^

**Figure 1:**
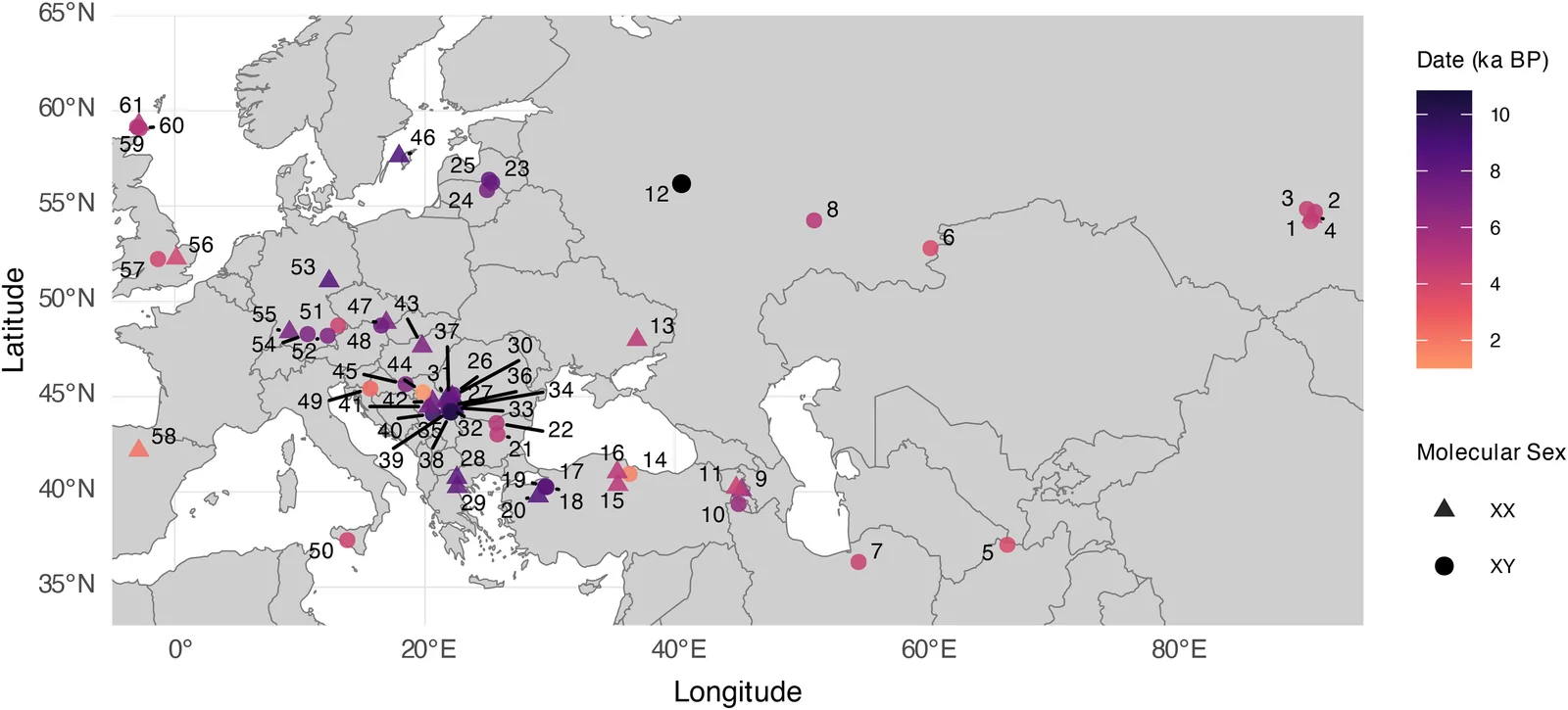
Geographic and temporal distribution of the ancient individuals included in the study Each point represents an individual, positioned according to its sampling location. Point color indicates the estimated date in thousands of years before present (ka BP), with darker colors representing older individuals. The individual older than 30 ka BP (Sample 12) is shown in black and was excluded from the color scale. Points’ shape denotes molecular sex. Individuals are numbered from east to west (Table S1). To improve clarity of the main geographic pattern, one sample (Sample 62, from the USA) is not shown in this panel (check Figure S1).

As maintaining CpG-level estimation was essential for our downstream analyses, we quantified the decay of autocorrelation among methylation levels of neighboring CpGs with increasing distance using modern whole-genome bisulfite sequencing data (WGBS). The strongest correlation could be found within windows of 10–25 CpGs (Figure S3). Beyond this range, correlations declined steadily, suggesting that larger windows begin to incorporate CpGs from distinct regulatory elements. Based on our observations, we selected windows of 10, 15, 20, and 25 CpGs for downstream analyses. For individual methylomes, the expected bimodal distribution characteristic of mammalian DNA methylation, with enrichment near fully unmethylated (0) and fully methylated (1) states^35^^,^^36^ was observed consistently across all 22 autosomes, thus indicating that the reconstructed F-values reflect true methylation structure rather than random noise (Figures S4–S7). CpG islands overlapping with the 40k CpG panel of the mammalian methylation array^17^ also displayed pronounced hypomethylation (mean ≈0.11–0.14), consistent with canonical regulatory signatures observed in modern data for which the sites were selected (Figure S8).

We further evaluated whether reconstructed methylomes follow expected inter-individual patterns and that broad biological signals can be retained. Principal component analysis (PCA) on a set of 4,000 X chromosome-linked CpGs associated with genetic sex^37^ clearly separated genetically male and female individuals in our ancient dataset after restricting the analysis to samples with a mean depth of at least 10X for the X chromosome (Figure S9). This filtering step excluded 10 individuals, comprising two females and eight males. Notably, before the depth filtering, the two low-coverage female samples clustered with the male individuals (Figure S10), while applying the 10X threshold resulted in clear separation between males and females. This result demonstrates that low sequencing depth is insufficient for reliable DNA methylation analysis and that relying on low-depth genomes can obscure true biological signals, highlighting the necessity of adequate depth for meaningful interpretation of reconstructed ancient methylomes, consistent with the results of Çoğoğlu et al. (2024).^38^

Despite these validations, both previous work^25^^,^^28^^,^^31^^,^^32^ and our own analyses (Figures S11–S14; Table S2) show that raw DamMet F-values are not directly suitable for age prediction. Applying modern epigenetic clocks to these estimates resulted in poor age-prediction performance, with no consistent improvement across CpG window sizes or clock models (Figures S11–S14). Notably, poor performance persisted after incorporating anthropological age uncertainty through interval-aware Monte Carlo analyses (Table S2), indicating that the limited predictive accuracy is not solely driven by imprecision in age-at-death estimates. Moreover, absolute age estimates were poor and rarely fell within reported anthropological age ranges, indicating a systematic bias between damage-derived F-values and modern DNA methylation levels, necessitating a correction step prior to downstream analyses, as proposed by Liu and colleagues.^33^

### Correction of reconstructed methylomes

Following Liu et al.,^33^ we applied a correction approach previously introduced for ancient horses in order to reduce potential bias in ancient methylation estimates and improve their correlation with methylation patterns observed in modern data. After implementing the correction step, Spearman correlation (*R*) with modern blood-derived array methylomes increased substantially for all CpG window sizes (*R* = 0.56–0.79, 10 CpGs; *R* = 0.64–0.80, 15 CpGs; *R* = 0.70–0.82, 20 CpGs; *R* = 0.73–0.83, 25 CpG; *p*-value <0.001 for all, see Figure 2), while PCA on X-linked CpGs still captured the same patterns as prior to correction (Figures S15 and S16).

**Figure 2:**
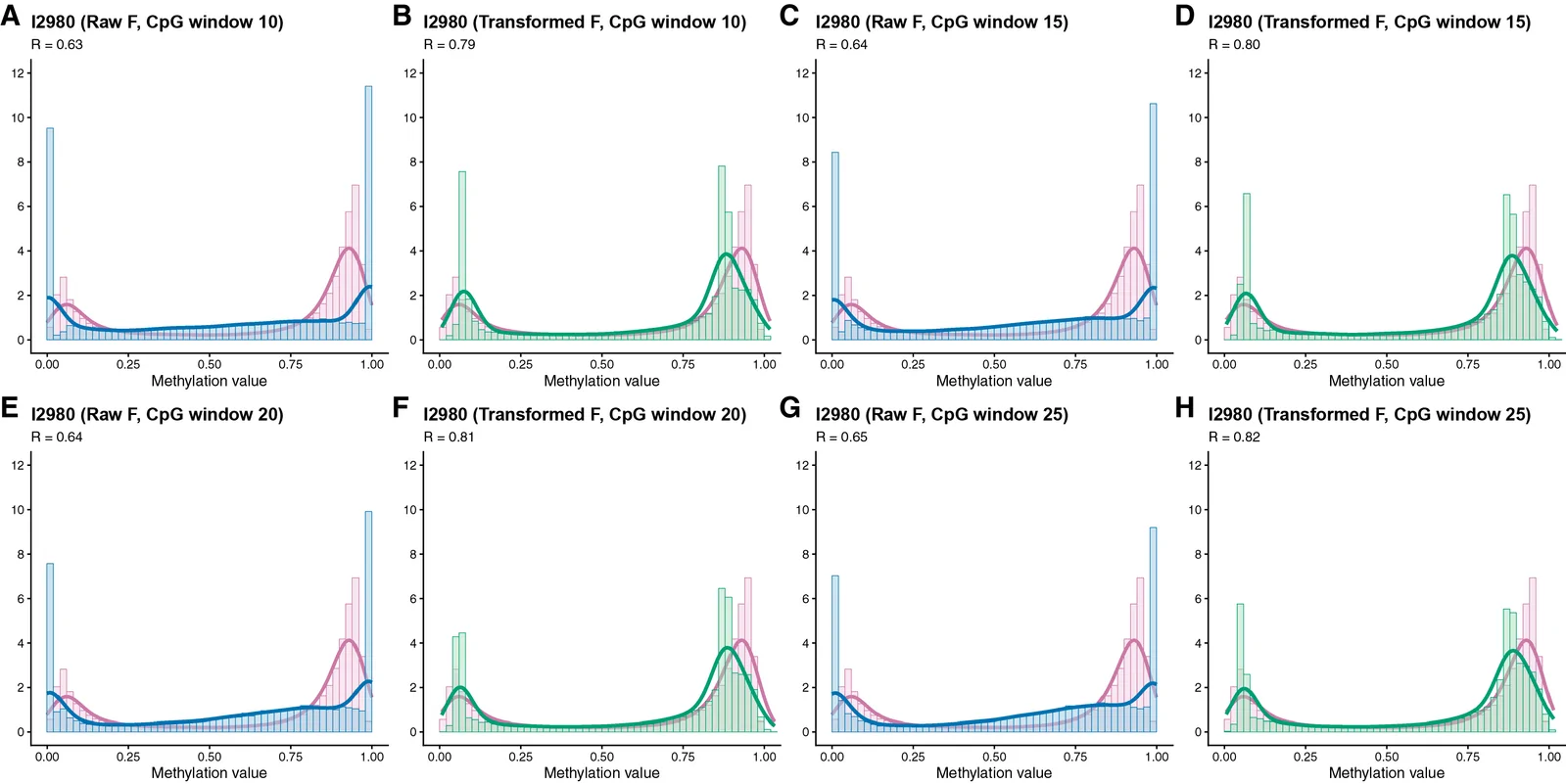
Distribution of methylation values across CpG window sizes before and after correction Histograms show the distribution of methylation values for sample “I2980” using 10- (A and B), 15- (C and D), 20- (E and F), and 25-CpG (G and H) windows. For each window size, the left panel (A, C, E, and G) shows raw DamMet F-values, whereas the right panel (B, D, F, and H) shows the corresponding transformed methylation (F) values. Overlaid density curves show raw DamMet F-values (blue), transformed methylation (F) values (green), and mean methylation values across *n* = 336 modern blood-derived samples (purple). Across all window sizes, correction shifts ancient methylation distributions toward that of the modern reference.

Although Spearman correlation was slightly higher in larger CpG windows (for example, from *R* = 0.79 at 10 CpGs to *R* = 0.82 at 25 CpGs in sample I2980 (*p*-value <0.001 for both), as shown in Figure 2), these gains were accompanied by only minor changes in overall distributional similarity. Kolmogorov-Smirnov distances differed only marginally across window sizes (0.23, 0.21, 0.20, 0.19, respectively, with *p* < 0.001 for all), indicating that the improvement from additional smoothing was limited. Therefore, we focused on the 10-CpG window to evaluate whether these corrected methylation value estimates could improve performance in downstream age-at-death prediction models.

### Application of modern epigenetic clocks after correction

To test the direct applicability of established modern epigenetic clocks (with reported mean absolute error of 2–7 years) on the transformed ancient methylomes, five different models were evaluated: (1) the BBT-APM clock,^23^ a tissue-matched model with a small (7) CpG panel; (2) the 37-CpG model,^39^ which was specifically trained on skeletal samples including petrous bone; (3&4) two broadly calibrated pan-mammalian/pan-tissue models with large (328 and 793, respectively) CpG panels (UniversalPanMammalianClock1 and UniversalPanMammalianClock2),^18^ and; (4) the widely used Horvath clock based on age-dependent DNA methylation profiles at 353 CpG sites.^12^

As anthropological age-at-death is typically given as an interval rather than precise point estimates, conventional point-based error metrics such as mean absolute error (MAE) or root-mean-square error (RMSE) are not well suited as primary performance measures for our analysis. Instead, individual clock performance was evaluated using an interval-aware Monte Carlo Spearman framework that explicitly accounts for age uncertainty by repeatedly sampling plausible ages within the reported age-at-death ranges and by quantifying the consistency of relative age ordering across ancient individuals (Table S3).

Our initial findings for the 10-CpG window (Figure 3) showed that the two universal pan-mammalian clocks exhibited a weak but consistent monotonic correlation with anthropological age-at-death, with a median Monte Carlo Spearman (*R*_MCS_) correlation of 0.25 (95% CI: 0.16–0.35) for Universal Pan Mammalian Clock 1, while Universal Pan Mammalian Clock 2 showed a slightly lower value with 0.21 (95% CI: 0.13–0.29). In contrast, for the BBT-APM clock a consistent negative correlation with the reported age of −0.14 (95% CI: −0.22 to −0.04) was found, while predictions based on the Horvath clock showed a lack of correlation (0.03; 95% CI: −0.05–0.10), and for the 37-CpG model only a marginal correlation was detected (0.11, 95% CI: 0.02–0.19).

**Figure 3:**
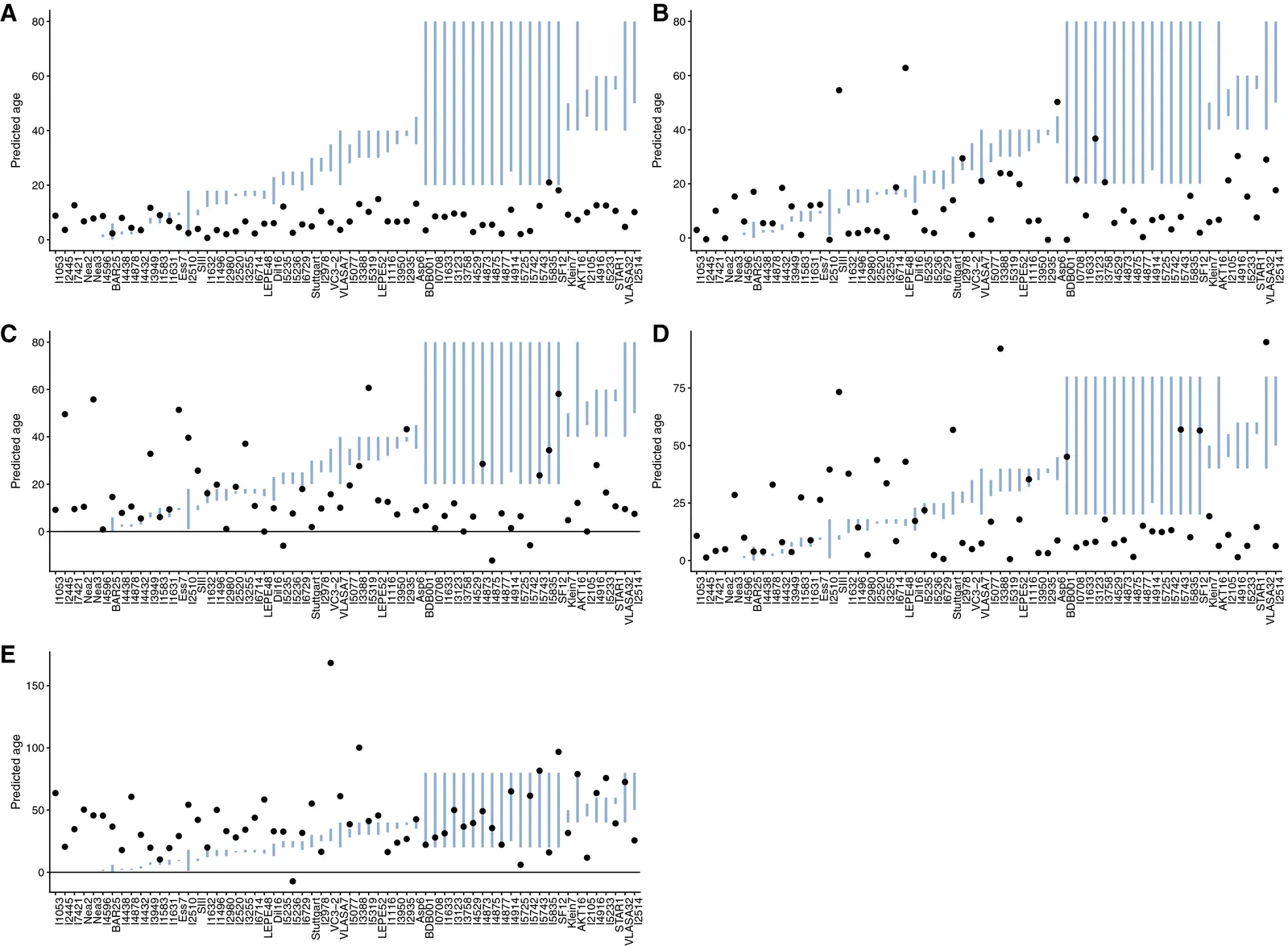
Performance of epigenetic clocks on corrected ancient methylomes (CpG window 10) Panels show results for (A) UniversalPanMammalianClock1, (B) UniversalPanMammalianClock2, (C) BBT-APM, (D) the Horvath clock, and (E) the 37-CpG model. Vertical lines (blue) indicate anthropological age-at-death ranges, and points denote clock-derived predicted ages.

Despite partial preservation of the relative order of ages for some clocks, absolute age estimates were largely inconsistent with anthropological estimates. For the 10-CpG window, only a small fraction of predictions fell within the reported age ranges (8.1–22.6%), with the highest proportion observed for the 37-CpG model. However, its predictions remained biased toward adult ages (overall median = 27 years, subadult median = 23 years, and adult median = 30 years). Moreover, absolute median distances to the nearest anthropological age boundary were approximately 11–13 years across all five clocks. Thus, even when partial age ordering was recovered, predicted ages remained inaccurate in absolute terms, and they deviated substantially from anthropological age ranges. Analyses using larger CpG windows (15, 20, and 25) produced qualitatively similar results (Figures S17–S19; Table S3).

Compared to the analyses conducted on the uncorrected F-values, the correction step seems to have produced model- and window-dependent changes rather than a systematic improvement in clock performance (Figure S20) and did not produce reliable absolute age estimates.

Because of the limited performance of the established clocks, we further tested whether age-at-death could be inferred by fitting a regression model on modern blood-derived methylation array data and applying it to reconstructed ancient methylomes. Unfortunately, this approach also failed to produce meaningful age estimates, with predicted ages being uniformly negative and showing no meaningful association with anthropological age-at-death estimates (Figure S21). These results further indicate that continuous age regression is not directly transferable to reconstructed ancient methylomes.

Taken together, these results suggest that age-associated signals are not entirely absent from reconstructed ancient methylomes, but that their translation into accurate age estimates is likely constrained by the differences between reconstructed ancient methylomes and the data on which the modern epigenetic clocks were trained. Modern clocks are typically developed using precise DNA methylation measurements from fresh tissues,^22^ conditions that differ fundamentally from damage-derived methylation inference in ancient DNA. In addition, the limited precision achievable at a sequencing depth around 10X further impedes reliable age prediction, as accurate methylation inference at the CpG level has been shown to require substantially higher coverage than usually available.^29^ Consequently, reliable age prediction from methylomes might require models explicitly trained on high-precision ancient methylation data, rather than the application of existing clocks or minor adaptation of modern epigenetic clocks. We therefore tested if it is possible to reframe age inference as a binary classification problem rather than a continuous prediction task.

### Adult-subadult classification

While attempts at direct age prediction failed so far, we tested if enough age-related information could be retrieved from our reconstructed methylomes to distinguish between broad age classes. We therefore divided our data based on the anthropological age estimates into adults and subadults, using an arbitrary cut-off of 18 years. To further reduce ambiguity in anthropological age-at-death estimates, we restricted the analysis to individuals with clearly defined age ranges, excluding samples with mean estimates overlapping 15–25 years. This resulted in 50 individuals (16 subadults and 34 adults). Classification was performed using an elastic net-regularized logistic regression model trained on modern blood-derived methylation array data. When evaluated by cross-validation on the modern dataset, the model achieved perfect classification between the two classes, confirming that this age contrast is readily learnable under conditions of precise age labels and high-quality methylation quantification.

However, when evaluated on our ancient samples, across all CpG window sizes, overall classification accuracy remained limited, with total accuracy never exceeding 60% (Figure 4; Table S4). Apparent high accuracies observed for specific age classes in some models reflected strong classification bias rather than robust age classification, as in the modern dataset. In particular, CpG windows 10 and 25 showed pronounced asymmetry in class assignment, with the 10-CpG window preferentially classifying individuals as adults (adult accuracy 76%, subadult accuracy 25%) and the 25-CpG window exhibiting the opposite trend, over-classifying subadults (accuracy for subadults = 75%, and adults = 32%). Although these windows showed modest overall performance (accuracy = 60% and 46%, respectively), Fisher’s exact tests indicated no significant association between predicted and anthropological age classes (odds ratios: 1.1 and 1.4, both *p* > 0.7), showing that apparent class-specific performance was driven by systematic bias rather than reliable age classification. Moreover, the 15-CpG window showed the most balanced performance across age classes, with similar accuracies for adults (59%) and subadults (44%), with an overall accuracy of 54%, yet again not different from a random assignment. The 20-CpG window performed poorly for both classes, with an overall accuracy of only 36% and no significant class association as well.

**Figure 4:**
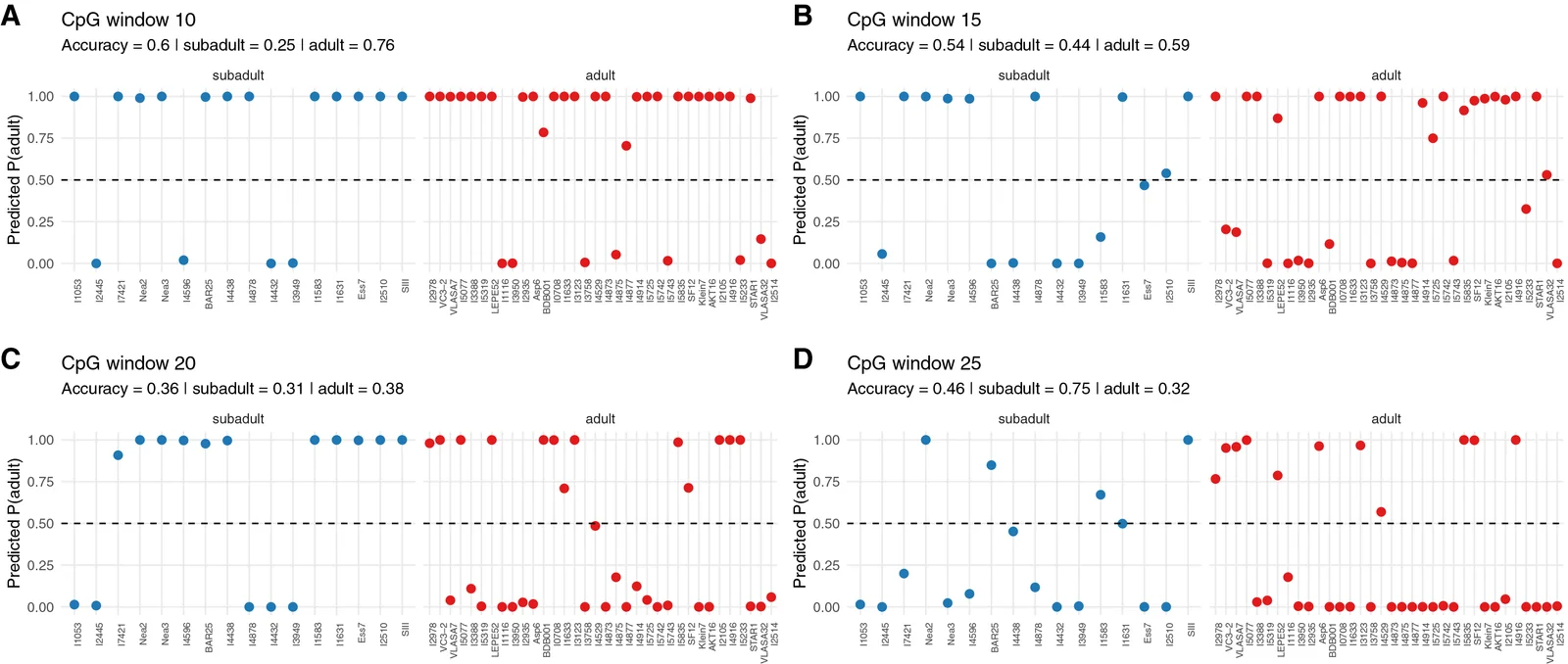
Predicted probability of subadult/adult classification for ancient individuals across different CpG windows Predicted classfication probabilities are shown for ancient individuals using (A) 10-, (B) 15-, (C) 20-, (D) 25-CpG windows. Points represent individual samples, colored by anthropological age classes (subadult; blue, adult; red). Dashed horizontal lines indicate the classification threshold (*p* = 0.5).

These opposing class-specific error patterns indicate that apparent classification success is largely driven by systematic, window-dependent biases rather than by a stable and biologically meaningful age signal. Consistent with this interpretation, Fisher’s exact tests revealed no significant association between predicted and anthropological age classes for any of the CpG windows, indicating that observed accuracies do not exceed chance expectations. Although results varied across window sizes, none yielded statistically supported age classification.

We further tested whether incorporating ancient samples into model training could stabilize performance by training mixed models combining modern and ancient individuals. Model evaluation was assessed through *k*-fold cross-validation, in which folds were defined exclusively on the ancient samples. In line with previous results, this approach did not lead to consistent performance improvement and instead showed instability across folds and window sizes (Table S5), reinforcing the conclusion that limited ancient sample size combined with heterogeneous methylation signal properties largely constrains reliable, supervised learning.

Finally, to address whether the poor performance observed so far was driven by the reliance on modern training data, we trained an additional classifier solely on ancient individuals. Given the limited size of our ancient dataset, the predictors were restricted to the 37 sites of the 37-CpG model. This helps to improve the sample-to-predictor ratio and to try to reduce the risk of overfitting by relying on a pre-selected set of features (i.e., CpGs). Additionally, the 37-CpG model was trained and tested on skeletal tissues, including petrous bone, and hence consists of CpGs that were previously associated with age in skeletal tissue.^39^ However, the classifier predicted all test samples as adults, showing no sensitivity to identify subadults. Thus, training only using ancient data and restricting the analysis to a smaller CpG panel both failed to recover a sufficiently stable age-associated signal for binary classification.

Taken together, these results suggest that the main constraint is not the modeling strategy itself, but rather the inability to recover a sufficiently stable and informative age-associated DNA methylation signal from reconstructed ancient methylomes.

### Sensitivity of age prediction to DNA methylation inference error and sample size

We performed two additional complementary analyses using the modern methylation array data (336 samples) in order to assess whether the limited performance observed in the ancient dataset could be explained mainly by its small sample size or by imprecision in the site-specific ancient DNA methylation inference. First, we progressively perturbed the DNA methylation values by iteratively adding random noise (±10%, 20%, 30%, 40%) prior to predicting the age of each individual using the UniversalPanMammalianClock2. Age prediction performance deteriorated consistently with increasing noise, with RMSE increasing from 2.6 years in the original dataset to 8.8, 13.2, 17.3, and 21.5 years, respectively (Figure S22). This pattern shows that age prediction is highly sensitive to even moderate imprecision in the DNA methylation signal, consistent with the requirement for high sequencing depth for reliable methylation-based age prediction in modern studies.^21^

We next repeatedly downsampled the modern dataset to match the size and class distribution of the ancient dataset (24 subadults and 38 adults). Across 1000 iterations, the mean RMSE values for the downsampled datasets remained similar to the RMSE values obtained using the full modern dataset, even when looking into each age class separately (Table S6). Thus, reducing the dataset to 62 individuals had little effect on the prediction error, whereas the perturbation of the methylation values produced a progressive loss of accuracy.

While the simulated perturbations do not necessarily represent direct estimates of error in the reconstructed ancient methylomes, these results suggest that the weak performance of age prediction observed in the ancient dataset is less likely to be explained solely by sample size, rather than imprecision of site-specific inferred methylation signals being a major limiting factor.

### Conclusions

This study highlights both the promise and current limitations of ancient epigenetic age estimation when applied to ancient human genomes. By systematically evaluating reconstructed ancient methylomes before and after applying a correction framework adapted from Liu et al. (2023), we show that correction improves similarity between human ancient and modern methylation data and suggests that limited age-related structure could be recovered. However, in contrast to results reported for modern or historical datasets, this correction was not sufficient to allow for reliable age-at-death estimation from ancient human genomes, neither as continuous predictions nor as binary classifications. Predicted ages showed limited consistency with anthropological age estimates and no CpG window size produced statistically supported age classification. Together, these findings indicate that while correction is a necessary step for mitigating systematic biases inherent to damage-derived methylation inference,^33^ it does not fully reconcile the fundamental differences between reconstructed ancient methylomes and the assumptions under which modern epigenetic clock models are trained. Consequently, reliable age-at-death inference from ancient human genomes, even at sequencing depths up to ∼35X, as of now, remains out of reach. Future progress will hinge on larger and more diverse ancient datasets, systematic comparisons across skeletal tissues and developmental stages, and different populations, along with the development of bone-specific reference methylomes. Closer collaboration with forensic epigenetics and the integration of emerging methylation profiling technologies, such as array-based assays, bisulfite sequencing, or nanopore sequencing, may ultimately enable the development of age-prediction models explicitly optimized for ancient DNA and, in the longer term, strengthen demographic reconstructions of past populations and revive paleodemographic research.

### Limitations of the study

This study shows that ancient epigenetic age estimation is currently limited mostly by both data availability and methodological constraints. However, these results must be interpreted in the light of several limitations inherent to biomolecular data. First, the number of ancient individuals available for analysis remains limited, restricting statistical power and the ability to train or robustly evaluate supervised models across age categories. This limitation is particularly relevant for classification and cross-validation analyses, as small and imbalanced datasets increase sensitivity to individual samples and preprocessing choices. Second, the precision of damage-derived methylation estimates is constrained by sequencing depth, which limits the reliability of site-specific methylation level estimation and thereby the precision needed for age-at-death estimation at coverage levels typical of ancient genomes. Third, most ancient genomes analyzed in this study were derived from petrous bone, a skeletal element whose inner portion forms early in life and is thought to exhibit limited remodeling and low turnover rates.^40^ As a consequence, methylation patterns recovered from petrous bone may reflect an effectively younger or more homogenized biological age than the true individual age at death. We attempted to address this by evaluating and using the 37-CpG model proposed by Gopalan et al. 2019, which was developed using skeletal tissues, including petrous bone. However, this did not improve age prediction or classification. Additionally, PCA based on corrected age-associated CpGs showed a general overlap between petrous and non-petrous samples, with no clear separation according to skeletal tissue source (Figure S23). These results suggest that the skeletal source alone is unlikely to explain the poor age estimates and that the reconstructed ancient methylomes may instead lack the sensitivity required to capture subtle age-associated differences. However, although the limited number of samples derived from other skeletal elements (teeth and long bones) did not show improved age inference in our dataset, this precludes strong conclusions regarding tissue-specific performance. Finally, additional biological factors may also have contributed to the limited performance observed here. This includes population-specific genetic background and mismatch between training and test datasets.^41^^,^^42^ However, the ancient individuals analyzed here originated from diverse geographic regions and time periods, and poor performance persisted independent of the approach and training datasets. Population heterogeneity may hence have contributed additional variability but is unlikely to fully explain the consistent inability to recover age-associated methylation signals. Moreover, a recent study on *Peromyscus* indicated that parental relatedness can also affect epigenetic age estimation,^43^ but its relevance to humans remains unknown.

## Resource availability

### Lead contact

Further information and requests for resources should be addressed to and will be fulfilled by the lead contact, Youssef Tawfik ytawfik@uni-mainz.de.

### Materials availability

This study did not generate new unique reagents.

### Data and code availability

• All genomic data used in this study are available from the sources cited in the STAR Methods section or are available upon request from the Allen Ancient Genome Diversity Project (https://reich.hms.harvard.edu/ancient-genome-diversity-project).
• Code written as part of the current study is available via Github (https://github.com/tawfikyoussef/EpiAaD).
• Any additional information required to reanalyze the data reported in this paper is available from the lead contact upon request.

## Acknowledgments

We would like to thank Nicoletta Zedda, Laura Winkelbach, Maxime Brami, Joan Barau, Miguel Andrade, and Daniel Wegmann for the helpful discussions, Katterinne Mendez for assistance, and the GenEvo consortium for support. We thank Shyamalika Gopalan for sharing the coefficients for the 37-CpG model. We thank David Reich for access to unpublished genomes from the Allen Ancient Genome Diversity Project (AAGDP). Parts of this research were conducted using the supercomputer MOGON 2 at Johannes Gutenberg University of Mainz (hpc.uni-mainz.de). This work was funded by the 10.13039/501100001659Deutsche Forschungsgemeinschaft (10.13039/501100001659DFG, 10.13039/501100001659German Research Foundation) – GRK2526/1 – Projectnr. [407023052]; Y.D. was funded by the ERC Advanced Grant [788616] (‘YMPACT’), awarded to Volker Heyd; L.O. was funded by the ERC Synergy Grant [101071707] (‘Horsepower’).

## Author contributions

Conceptualisation: J.Bu., Y.D.; methodology, formal analysis, and investigation: Y.T. with input from J.Bl.; writing – original draft: Y.T. with input from J.Bl., L.O.; Writing – review and editing: all authors.

## Declaration of interests

The authors declare that they have no competing interests.

## Declaration of generative AI and AI-assisted technologies in the manuscript preparation process

During the preparation of this work, the authors used ChatGPT to assist in language proofreading. After using this tool, the authors reviewed and edited the content as needed and take full responsibility for the content of the published article.

## STAR★Methods

### Key resources table

**Table:** 

REAGENT or RESOURCE	SOURCE	IDENTIFIER
**Deposited data**		

Allen Ancient Genome Diversity Project	Allen Ancient Genome Diversity Project^44^	https://reich.hms.harvard.edu/ancient-genome-diversity-project

**Software and algorithms**		

Sambamba	Tarasov et al.^45^	https://lomereiter.github.io/sambamba/
GATK (v3.8)	McKenna et al.^46^	https://gatk.broadinstitute.org/hc/en-us
ATLAS	Link et al.^47^	https://atlaswiki.netlify.app/
DamMet	Hanghøj et al.^29^	https://github.com/KHanghoj/DamMet
R (v4.5.3)	Team et al.^48^	https://www.r-project.org/
Python (v3.10.12)	Python Software Foundation	https://www.python.org/
Analysis scripts	This study; GitHub	https://github.com/tawfikyoussef/EpiAaD

### Experimental model and study participant details

Our dataset consists of 62 publicly available ancient human genomes (Table S1; Figures 1 and S1).

### Method details

#### Whole genome sequencing data collection

We assembled a dataset of 62 ancient human genomes sequenced to high average depth-of-coverage, which were either publicly available,^49^^,^^50^^,^^51^^,^^52^^,^^53^^,^^54^^,^^55^^,^^56^^,^^57^^,^^58^^,^^59^^,^^60^^,^^61^^,^^62^^,^^63^^,^^64^^,^^65^^,^^66^^,^^67^^,^^68^^,^^69^ or available upon request from the Allen Ancient Genome Diversity Project^44^ (Table S1). Three criteria guided sample selection: (i) an average sequencing depth greater than 10X, (ii) reports of USER treatment in library preparation, ensuring reliable DNA methylation inference,^28^^,^^29^ and (iii) the availability of an anthropological age estimate, at minimum a categorical assignment. Applying these filters produced 24 subadult and 38 adult individuals. Since DNA methylation is tissue-specific, we aimed to minimise variability by relying primarily on petrous bone samples. However, due to sample limitations, six genomes were derived from other skeletal elements (Table S1) and were still added to our dataset.

#### Ancient DNA processing and methylation inference

Published genomes were obtained from their respective ENA repositories in BAM format, further additional unpublished data for previously published individuals were provided by David Reich’s lab (see Table S1 for details). After the removal of PCR duplicates with sambamba,^45^ all genomes were re-aligned around a set of known SNPs and InDels using GATK 3.8.^46^ Variant detection was performed with ATLAS^47^ as described in Marchi et al. 2022.^55^

To infer DNA methylation values, we used the DamMet statistical package (v1.0.4),^29^ with default parameters in both steps, the “-R” flag to indicate the read groups from the BAM files, excluding any truncated read groups, the “-E” flag to exclude sites with true variants, a maximum CpG window “-N” of 10, 15, 20, and 25, and the “-skip_empty_cpg” parameter.

#### Ancient methylome structure assessment

We evaluated two properties of DNA methylation structure. We first confirmed the expected bimodal distribution of F-values inferred by DamMet by plotting genome-wide methylation profiles per autosome, per sample. We then evaluated whether age-related CpGs – as a representative subset of focal CpG sites – retain coherent methylation patterns across the CpG window sizes used in DamMet. Using four WGBS samples from the ENCODE Project^70^ (experiments accessions: ENCSR012TGL, ENCSR579AXB, ENCSR307KDA, ENCSR766GKN), we calculated the correlation between the methylation level of each age-related focal CpG and the mean methylation of its neighboring CpGs in windows from 2 to 50 CpGs (step size 2). The correlation trend was used to assess the suitability of CpG windows for DamMet inference.

#### Sex estimation

We inferred molecular sex using DNA methylation patterns along the X chromosome. A panel of ∼4,000 CpGs from the watermelon R package (v.2.8.0^37^) was extracted, and principal component analysis (R function prcomp, in R package pROC v.1.18.5) was used to visualise separation between males and females, reflecting sex-specific methylation differences.

#### Implementation of the correction step on ancient data

We implemented the two-step methylation transformation described by Liu et al. (2023). For each CpG site, we constructed predictor variables including the DamMet F-value, its square, sequencing depth, and the interaction between F and depth. A Random Forest (RF) regression model was then trained to predict modern reference methylation values, followed by a linear regression to rescale the RF predictions to match the distribution observed in modern data. The linear regression-adjusted values were used as the final transformed DNA methylation values.

As modern references, we used publicly available methylation datasets generated by the Mammalian Methylation Consortium on the HorvathMammalMethylChip40 array,^17^ specifically GSE184221 and GSE184215,^18^ with a total of 336 samples. For each CpG site represented in these datasets, we calculated the mean methylation value across all modern samples, which served as the baseline for transformation. Following Liu and colleagues (2023), an exponent parameter (X) was optimised by minimising prediction error of the RF model, and the optimised value was used to transform the modern reference methylation values. The RF model was assessed and tuned using repeated random 90/10% train-test split at the sample level, based on these evaluations, 200 trees were selected for the final model in the ranger package (v0.17.0), with mtry = 2. Age-associated CpGs (from the reported four modern epigenetic clocks) were set aside during training, as our goal was to evaluate their performance independent of age association. A custom R script was written to reproduce the transformation workflow of Liu et al. (2023), adapted to our dataset and analysis framework.

We then compared ancient methylation estimates with the corresponding modern reference mean methylation values for each CpG window size, to evaluate the effect of the correction on the overall distribution of methylation values. Differences between the two distributions were evaluated using a two-sample Kolmogorov-Smirnov test implemented with the base R function ks.test(), and the Kolmogorov-Smirnov statistic and corresponding *p*-value were reported for each window size.

#### Application of modern epigenetic clocks

We tested the performance of published epigenetic clocks on reconstructed ancient methylomes, before and after correction. The BBT-APM clock,^23^ the 37-CpG model,^39^ and the UniversalPanMammalianClock1 and UniversalPanMammalianClock2,^18^ where each published model was reimplemented in custom Python scripts following mathematical specifications of the original authors. In addition, the Horvath clock^12^ was used in R using the methylclock package (v1.8.0).^16^ Clock performance was assessed by comparing predicted ages to anthropological age estimates using three complementary metrics. First, association was quantified using a Monte Carlo Spearman correlation approach, in which ages were randomly sampled from the reported anthropological age intervals and Spearman’s rank correlation between predicted and sampled ages was calculated across iterations. Second, we calculated the percentage of predictions falling within the reported anthropological age intervals. Third, for predictions falling outside the interval, we quantified the distance to the nearest interval boundary.

### Quantification and statistical analysis

#### Developing an ancient epigenetic clock

An elastic net (EN) regression model was trained to predict chronological age from modern DNA methylation values at a predefined panel of age-associated CpGs corresponding to the union of the CpGs used in the four modern epigenetic clocks evaluated in this study. Modern methylation beta values and sample ages were obtained from the same Mammalian Methylation Consortium datasets described above. DNA methylation matrices were subset to CpGs present in both the age-associated panel and the modern data, standardised by *Z* score scaling, in which the scaling parameters were later retained for application to ancient data.

Model training was performed using glmnet (v5.0^71^) with a Gaussian family. The EN mixing parameter (alpha) was set to 0.5, and model regularisation was selected using 10-fold cross-validation (CV). Coefficients corresponding to lambda.1se were retained for prediction. A fixed random seed was used.

Clock performance was assessed by comparing predicted ages with anthropological age estimates using three complementary metrics. First, association was quantified using an interval-aware Monte Carlo Spearman correlation approach. For each of 10000 iterations, one age was sampled independently for each individual from a uniform distribution bounded by the minimum and maximum anthropological age estimates, and Spearman’s rank correlation was calculated between the sampled and predicted ages. The median correlation across iterations was reported together with the 2.5th and 97.5th percentiles. Spearman correlation with the mean anthropological age estimate was also calculated separately. Second, we calculated the percentage of predictions falling within the reported anthropological age intervals. Third, for predictions falling outside these intervals, we quantified the distance to the nearest interval boundary.

#### Adult-subadult classification

Binary age classification was performed using elastic net (EN) logistic regression implemented in glmnet^71^ with alpha set to 0.5 and a probability threshold of 0.5. Individuals were classified as subadults (≤18 years) or adults (>18 years). CpGs were restricted to the predefined age-associated panel abovementioned, and CpGs with zero variance across modern samples were excluded.

Ancient class labels were derived from anthropological age estimates, and individuals with ambiguous age assignment (between 15 and 25 years) were excluded, and remaining samples were assigned to subadult or adult classes. For each ancient dataset, corrected methylation F-values were reshaped into CpG x sample matrices, missing CpG values were imputed to the corresponding modern CpG mean, and values were scaled using modern centering and scaling parameters.

A baseline classifier was trained on modern samples using 10-fold CV, inverse-frequency class weights, and coefficients corresponding to lambda.1se. This model was applied to ancient samples to obtain predicted probabilities and class labels. Performance on ancient samples was evaluated using AUC, accuracy, and balanced accuracy. In addition, Fisher’s exact test was used to assess the association between observed and predicted age classes for each CpG window, with the corresponding odds ratio and *p*-value reported. To assess robustness, a domain-anchored analysis was additionally performed by training classifiers on combined modern and ancient data and evaluating performance using *k*-fold CV on ancient samples only (*k* = 5).

A classifier was trained using only ancient methylation profiles, with the same classification threshold and filtering explained above. The corrected methylation values were restricted to only the 37 sites from the 37-CpG model.^39^ The data were split into training and test datasets by sampling 80% of individuals separately within each class. CpGs with a zero-variance were removed and an EN logistic regression implemented in glmnet with an optimised alpha = 0 and a probability threshold of 0.5.

#### Sensitivity analysis of methylation noise and sample size

To assess the effect of methylation imprecision on age prediction, random noise was progressively added to modern methylation values using the base R jitter function. Four perturbation magnitudes were tested (0.1, 0.2, 0.3, and 0.4). For each original methylation value “M”, the perturbed value was defined as (M∗ = M + aU), where “a” is the perturbation magnitude and “U” is the sampled from a uniform distribution between −1 and 1. Resulting values were constrained to the valid methylation range of 0–1. Age predictions were generated for the original and each perturbed dataset using the UniversalPanMammalianClock2, and prediction performance was assessed using root-mean-square error (RMSE) between known and predicted ages.

We additionally performed repeated stratified downsampling of the modern dataset. We randomly sampled, without replacement, 24 individuals ≤18 years and 38 individuals >18 years in each of a 1000 iterations, matching the composition of the ancient dataset (*n* = 62). RMSE was calculated for each iteration for the complete downsampled dataset and separately for subadults and adults. Mean RMSE across the 1000 iterations was used to summarise prediction performance for each perturbation.

## Supplemental information

Document S1. Figures S1–S23

Table S1. Ancient sample metadata and sequencing information for all individuals included in this study

Table S2. Evaluation of modern epigenetic clocks on ancient samples before correction

Table S3. Evaluation of modern epigenetic clocks on ancient samples after correction

Table S4. Adult-subadult age classification performance for models trained on modern data, evaluated on ancient samples

Table S5. Adult-subadult age classification performance for models trained on combined ancient and modern data, evaluated on ancient samples

Table S6. Evaluation of the effect of progressive methylation noise on age-prediction accuracy in modern samples

## References

[1] McFadden C. (2021). The past, present and future of skeletal analysis in palaeodemography. Philos. Trans. R. Soc. Lond. B Biol. Sci..

[2] Boldsen J.L., Milner G.R., Ousley S.D. (2022). Paleodemography: From archaeology and skeletal age estimation to life in the past. Am. J. Biol. Anthropol..

[3] Diekmann Y., Smith D., Gerbault P., Dyble M., Page A.E., Chaudhary N., Migliano A.B., Thomas M.G. (2017). Accurate age estimation in small-scale societies. Proc. Natl. Acad. Sci. USA.

[4] Łukasik S., Bijak J., Krenz-Niedbała M., Sinika V. (2021). Paleodemographic analysis of age at death for a population of Black Sea Scythians: An exploration by using Bayesian methods. Am. J. Phys. Anthropol..

[5] Hoppa R.D., Vaupel J.W. (2002). Paleodemography: Age distributions from skeletal samples.

[6] Navega D., Costa E., Cunha E. (2022). Adult skeletal age-at-death estimation through deep random neural networks: A new method and its computational analysis. Biology.

[7] Bocquet-Appel J.-P., Masset C. (1982). Farewell to paleodemography. J. Hum. Evol..

[8] Mensforth R.P. (1990). Paleodemography of the Carlston Annis (Bt-5) Late Archaic skeletal population. Am. J. Phys. Anthropol..

[9] Bird A. (2002). DNA methylation patterns and epigenetic memory. Genes Dev..

[10] Moore L.D., Le T., Fan G. (2013). DNA methylation and its basic function. Neuropsychopharmacology.

[11] Bocklandt S., Lin W., Sehl M.E., Sánchez F.J., Sinsheimer J.S., Horvath S., Vilain E. (2011). Epigenetic predictor of age. PLoS One.

[12] Horvath S. (2013). DNA methylation age of human tissues and cell types. Genome Biol..

[13] Hannum G., Guinney J., Zhao L., Zhang L., Hughes G., Sadda S., Klotzle B., Bibikova M., Fan J.-B., Gao Y. (2013). Genome-wide methylation profiles reveal quantitative views of human aging rates. Mol. Cell.

[14] Zhang Q., Vallerga C.L., Walker R.M., Lin T., Henders A.K., Montgomery G.W., He J., Fan D., Fowdar J., Kennedy M. (2019). Improved precision of epigenetic clock estimates across tissues and its implication for biological ageing. Genome Med..

[15] Alfonso G., Gonzalez J.R. (2020). Bayesian neural networks for the optimisation of biological clocks in humans. bioRxiv.

[16] Pelegí-Sisó D., de Prado P., Ronkainen J., Bustamante M., González J.R. (2021). methylclock: a Bioconductor package to estimate DNA methylation age. Bioinformatics.

[17] Arneson A., Haghani A., Thompson M.J., Pellegrini M., Kwon S.B., Vu H., Maciejewski E., Yao M., Li C.Z., Lu A.T. (2022). A mammalian methylation array for profiling methylation levels at conserved sequences. Nat. Commun..

[18] Lu A.T., Fei Z., Haghani A., Robeck T.R., Zoller J.A., Li C.Z., Lowe R., Yan Q., Zhang J., Vu H. (2023). Universal DNA methylation age across mammalian tissues. Nat. Aging.

[19] Zoller J., Horvath S. (2024). MammalMethylClock R package: software for DNA methylation-based epigenetic clocks in mammals. Bioinformatics.

[20] Li C.Z., Haghani A., Yan Q., Lu A.T., Zhang J., Fei Z., Ernst J., Yang X.W., Gladyshev V.N., Raj K. (2023). Epigenetic predictors of species maximum lifespan and other life history traits in mammals. bioRxiv.

[21] Menendez Vazquez A., Katsanos D., Vasile M., Graham A., Dyster V., Kaveh S., Moqri M., Banila C. (2025). Epigenetic age predictors for non-invasive assessment of human skin. NPJ Aging.

[22] Teschendorff A.E., Horvath S. (2025). Epigenetic ageing clocks: statistical methods and emerging computational challenges. Nat. Rev. Genet..

[23] Correia Dias H., Manco L., Corte Real F., Cunha E. (2021). A Blood-Bone-Tooth Model for Age Prediction in Forensic Contexts. Biology.

[24] Briggs A.W., Stenzel U., Meyer M., Krause J., Kircher M., Pääbo S. (2010). Removal of deaminated cytosines and detection of in vivo methylation in ancient DNA. Nucleic Acids Res..

[25] Pedersen J.S., Valen E., Velazquez A.M.V., Parker B.J., Rasmussen M., Lindgreen S., Lilje B., Tobin D.J., Kelly T.K., Vang S. (2014). Genome-wide nucleosome map and cytosine methylation levels of an ancient human genome. Genome Res..

[26] Gokhman D., Lavi E., Prüfer K., Fraga M.F., Riancho J.A., Kelso J., Pääbo S., Meshorer E., Carmel L. (2014). Reconstructing the DNA methylation maps of the Neandertal and the Denisovan. Science.

[27] Gokhman D., Mishol N., de Manuel M., de Juan D., Shuqrun J., Meshorer E., Marques-Bonet T., Rak Y., Carmel L. (2019). Reconstructing Denisovan Anatomy Using DNA Methylation Maps. Cell.

[28] Hanghøj K., Seguin-Orlando A., Schubert M., Madsen T., Pedersen J.S., Willerslev E., Orlando L. (2016). Fast, Accurate and Automatic Ancient Nucleosome and Methylation Maps with epiPALEOMIX. Mol. Biol. Evol..

[29] Hanghøj K., Renaud G., Albrechtsen A., Orlando L. (2019). DamMet: ancient methylome mapping accounting for errors, true variants, and post-mortem DNA damage. GigaScience.

[30] Mathov Y., Rosen N., Leibson C., Meshorer E., Yakir B., Carmel L. (2025). RoAM: computational reconstruction of ancient methylomes and identification of differentially methylated regions. Genome Biol..

[31] Seguin-Orlando A., Donat R., Der Sarkissian C., Southon J., Thèves C., Manen C., Tchérémissinoff Y., Crubézy E., Shapiro B., Deleuze J.-F. (2021). Heterogeneous Hunter-Gatherer and Steppe-Related Ancestries in Late Neolithic and Bell Beaker Genomes from Present-Day France. Curr. Biol..

[32] Schmidt M., Maixner F., Hotz G., Pap I., Szikossy I., Pálfi G., Zink A., Wagner W. (2021). DNA methylation profiling in mummified human remains from the eighteenth-century. Sci. Rep..

[33] Liu X., Seguin-Orlando A., Chauvey L., Tressières G., Schiavinato S., Tonasso-Calvière L., Aury J.-M., Perdereau A., Wagner S., Clavel P. (2023). DNA methylation-based profiling of horse archaeological remains for age-at-death and castration. iScience.

[34] Affinito O., Palumbo D., Fierro A., Cuomo M., De Riso G., Monticelli A., Miele G., Chiariotti L., Cocozza S. (2020). Nucleotide distance influences co-methylation between nearby CpG sites. Genomics.

[35] Lister R., Pelizzola M., Dowen R.H., Hawkins R.D., Hon G., Tonti-Filippini J., Nery J.R., Lee L., Ye Z., Ngo Q.-M. (2009). Human DNA methylomes at base resolution show widespread epigenomic differences. Nature.

[36] Klughammer J., Romanovskaia D., Nemc A., Posautz A., Seid C.A., Schuster L.C., Keinath M.C., Lugo Ramos J.S., Kosack L., Evankow A. (2023). Comparative analysis of genome-scale, base-resolution DNA methylation profiles across 580 animal species. Nat. Commun..

[37] Wang Y., Hannon E., Grant O.A., Gorrie-Stone T.J., Kumari M., Mill J., Zhai X., McDonald-Maier K.D., Schalkwyk L.C. (2021). DNA methylation-based sex classifier to predict sex and identify sex chromosome aneuploidy. BMC Genom..

[38] Çokoğlu S.S., Koptekin D., Fidan F.R., Somel M. (2024). Investigating food production-associated DNA methylation changes in paleogenomes: Lack of consistent signals beyond technical noise. Evol. Appl..

[39] Gopalan S., Gaige J., Henn B.M. (2019). DNA methylation-based forensic age estimation in human bone. bioRxiv.

[40] Jørkov M.L.S., Heinemeier J., Lynnerup N. (2009). The petrous bone--a new sampling site for identifying early dietary patterns in stable isotopic studies. Am. J. Phys. Anthropol..

[41] Cruz-Gonzalez S., Okpala O., Gu E., Gomez L., Mews M., Vance J.M., Cuccaro M.L., Cornejo-Olivas M.R., Feliciano-Astacio B.E., Byrd G.S., Haines J. (2026). Methylation clocks fail to generalize across genetically admixed individuals. eLife.

[42] Meeks G.L., Scelza B., Asnake H.M., Prall S., Patin E., Froment A., Fagny M., Quintana-Murci L., Henn B.M., Gopalan S. (2025). Common DNA sequence variation influences epigenetic aging in African populations. Commun. Biol..

[43] Huynh-Dam K.-T., Jaeger C., Chatzistamou I., Horvath S., Kiaris H. (2026). Parental kinship influences global methylation and epigenetic age estimation in Peromyscus. Genetics.

[44] (2026). Allen ancient genome diversity project. https://reich.hms.harvard.edu/ancient-genome-diversity-project.

[45] Tarasov A., Vilella A.J., Cuppen E., Nijman I.J., Prins P. (2015). Sambamba: fast processing of NGS alignment formats. Bioinformatics.

[46] McKenna A., Hanna M., Banks E., Sivachenko A., Cibulskis K., Kernytsky A., Garimella K., Altshuler D., Gabriel S., Daly M., DePristo M.A. (2010). The Genome Analysis Toolkit: a MapReduce framework for analyzing next-generation DNA sequencing data. Genome Res..

[47] Link V., Kousathanas A., Veeramah K., Sell C., Scheu A., Wegmann D. (2017). ATLAS: Analysis Tools for Low-depth and Ancient Samples. bioRxiv.

[48] Team R.C. (2013). R: a language and environment for statistical computing. https://www.R-project.org/.

[49] Flegontov P., Altınışık N.E., Changmai P., Rohland N., Mallick S., Adamski N., Bolnick D.A., Broomandkhoshbacht N., Candilio F., Culleton B.J. (2019). Palaeo-Eskimo genetic ancestry and the peopling of Chukotka and North America. Nature.

[50] Gamba C., Jones E.R., Teasdale M.D., McLaughlin R.L., Gonzalez-Fortes G., Mattiangeli V., Domboróczki L., Kővári I., Pap I., Anders A. (2014). Genome flux and stasis in a five millennium transect of European prehistory. Nat. Commun..

[51] Gelabert P., Bickle P., Hofmann D., Teschler-Nicola M., Anders A., Huang X., Olalde I., Fournier R., Ringbauer H., Akbari A. (2023). Social and genetic diversity among the first farmers of Central Europe. bioRxiv.

[52] Gokhman D., Nissim-Rafinia M., Agranat-Tamir L., Housman G., García-Pérez R., Lizano E., Cheronet O., Mallick S., Nieves-Colón M.A., Li H. (2020). Differential DNA methylation of vocal and facial anatomy genes in modern humans. Nat. Commun..

[53] Lazaridis I., Alpaslan-Roodenberg S., Acar A., Açıkkol A., Agelarakis A., Aghikyan L., Akyüz U., Andreeva D., Andrijašević G., Antonović D. (2022). The genetic history of the Southern Arc: A bridge between West Asia and Europe. Science.

[54] Lazaridis I., Nadel D., Rollefson G., Merrett D.C., Rohland N., Mallick S., Fernandes D., Novak M., Gamarra B., Sirak K. (2016). Genomic insights into the origin of farming in the ancient Near East. Nature.

[55] Marchi N., Winkelbach L., Schulz I., Brami M., Hofmanová Z., Blöcher J., Reyna-Blanco C.S., Diekmann Y., Thiéry A., Kapopoulou A. (2022). The genomic origins of the world’s first farmers. Cell.

[56] Mathieson I., Lazaridis I., Rohland N., Mallick S., Patterson N., Roodenberg S.A., Harney E., Stewardson K., Fernandes D., Novak M. (2015). Genome-wide patterns of selection in 230 ancient Eurasians. Nature.

[57] Mathieson I., Alpaslan-Roodenberg S., Posth C., Szécsényi-Nagy A., Rohland N., Mallick S., Olalde I., Broomandkhoshbacht N., Candilio F., Cheronet O. (2018). The genomic history of southeastern Europe. Nature.

[58] Narasimhan V.M., Patterson N., Moorjani P., Rohland N., Bernardos R., Mallick S., Lazaridis I., Nakatsuka N., Olalde I., Lipson M. (2019). The formation of human populations in South and Central Asia. Science.

[59] Olalde I., Brace S., Allentoft M.E., Armit I., Kristiansen K., Booth T., Rohland N., Mallick S., Szécsényi-Nagy A., Mittnik A. (2018). The Beaker phenomenon and the genomic transformation of northwest Europe. Nature.

[60] Olalde I., Mallick S., Patterson N., Rohland N., Villalba-Mouco V., Silva M., Dulias K., Edwards C.J., Gandini F., Pala M. (2019). The genomic history of the Iberian Peninsula over the past 8000 years. Science.

[61] Rohland N., Mallick S., Mah M., Maier R., Patterson N., Reich D. (2022). Three assays for in-solution enrichment of ancient human DNA at more than a million SNPs. Genome Res..

[62] Sikora M., Seguin-Orlando A., Sousa V.C., Albrechtsen A., Korneliussen T., Ko A., Rasmussen S., Dupanloup I., Nigst P.R., Bosch M.D. (2017). Ancient genomes show social and reproductive behavior of early Upper Paleolithic foragers. Science.

[63] Wohns A.W., Wong Y., Jeffery B., Akbari A., Mallick S., Pinhasi R., Patterson N., Reich D., Kelleher J., McVean G. (2022). A unified genealogy of modern and ancient genomes. Science.

[64] Akbari A., Perry A., Barton A.R., Kariminejad M., Gazal S., Li Z., Zeng Y., Mittnik A., Patterson N., Mah M. (2026). Ancient DNA reveals pervasive directional selection across West Eurasia. Nature.

[65] Lipson M., Szécsényi-Nagy A., Mallick S., Pósa A., Stégmár B., Keerl V., Rohland N., Stewardson K., Ferry M., Michel M. (2017). Parallel palaeogenomic transects reveal complex genetic history of early European farmers. Nature.

[66] Günther T., Malmström H., Svensson E.M., Omrak A., Sánchez-Quinto F., Kılınç G.M., Krzewińska M., Eriksson G., Fraser M., Edlund H. (2018). Population genomics of Mesolithic Scandinavia: Investigating early postglacial migration routes and high-latitude adaptation. PLoS Biol..

[67] Rivollat M., Jeong C., Schiffels S., Küçükkalıpçı İ., Pemonge M.-H., Rohrlach A.B., Alt K.W., Binder D., Friederich S., Ghesquière E. (2020). Ancient genome-wide DNA from France highlights the complexity of interactions between Mesolithic hunter-gatherers and Neolithic farmers. Sci. Adv..

[68] Gelabert P., Bickle P., Hofmann D., Teschler-Nicola M., Anders A., Huang X., Hämmerle M., Olalde I., Fournier R., Ringbauer H. (2025). Social and genetic diversity in first farmers of central Europe. Nat. Hum. Behav..

[69] Lazaridis I., Patterson N., Anthony D., Vyazov L., Fournier R., Ringbauer H., Olalde I., Khokhlov A.A., Kitov E.P., Shishlina N.I. (2025). The genetic origin of the Indo-Europeans. Nature.

[70] Moore J.E., Purcaro M.J., Pratt H.E., Epstein C.B., Shoresh N., Adrian J., Kawli T., Davis C.A., Dobin A., ENCODE Project Consortium (2020). Expanded encyclopaedias of DNA elements in the human and mouse genomes. Nature.

[71] Friedman J., Hastie T., Tibshirani R. (2010). Regularization paths for generalized linear models via coordinate descent. J. Stat. Softw..

