# Supplementary material for: Promises and limitations of current ancient human epigenetic clocks
Source: iScience. 2026 Oct 28;29(11):118061. doi: 10.1016/j.isci.2026.118061 (PMC13733310; doi:10.1016/j.isci.2026.118061)
Supplement: Supplementary file 1 [file mmc1.pdf]

iScience, Volume *29*

Supplemental information

Promises and limitations of current ancient human epigenetic clocks

Youssef Tawfik, Yoan Diekmann, Ludovic Orlando, Joachim Burger, and Jens Blöcher

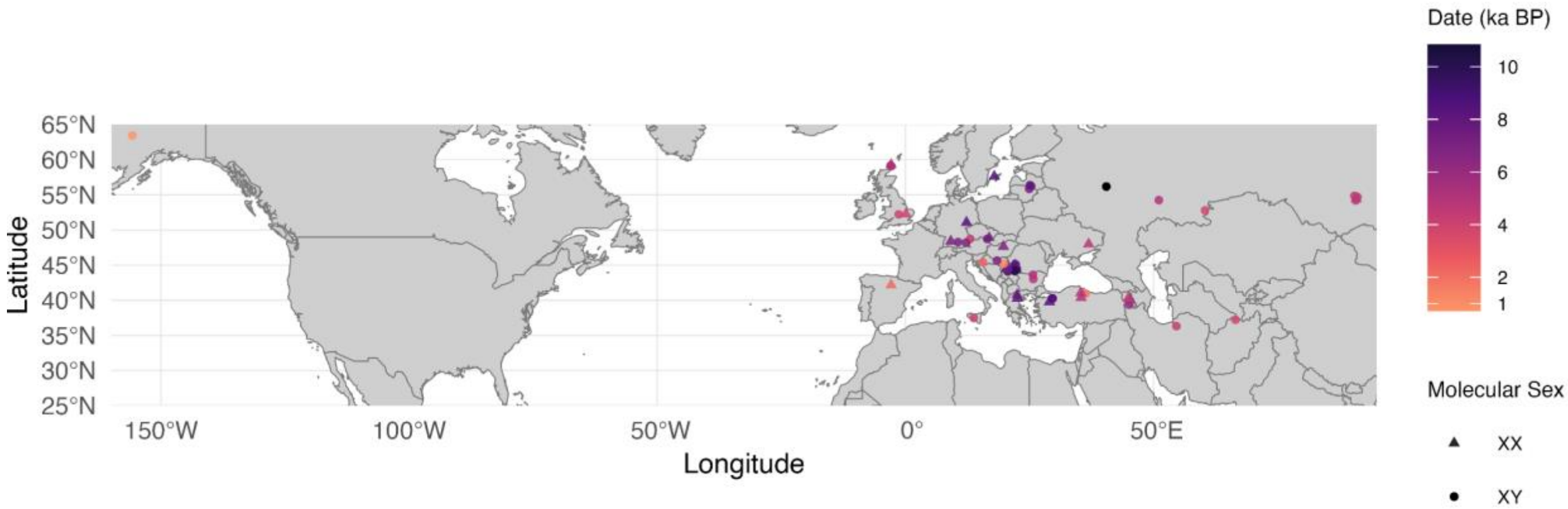

Supplementary Figure S1. Geographic and temporal distribution of all ancient individuals included in the study. Each point represents an individual, positioned according to its sampling location (Supplementary Table S1). Point color indicates the estimated date in thousands of years before present (ka BP), with darker colours representing older individuals. The single individual older than 30 ka BP is shown in black and excluded from the color scale. Points' shape denotes molecular sex.

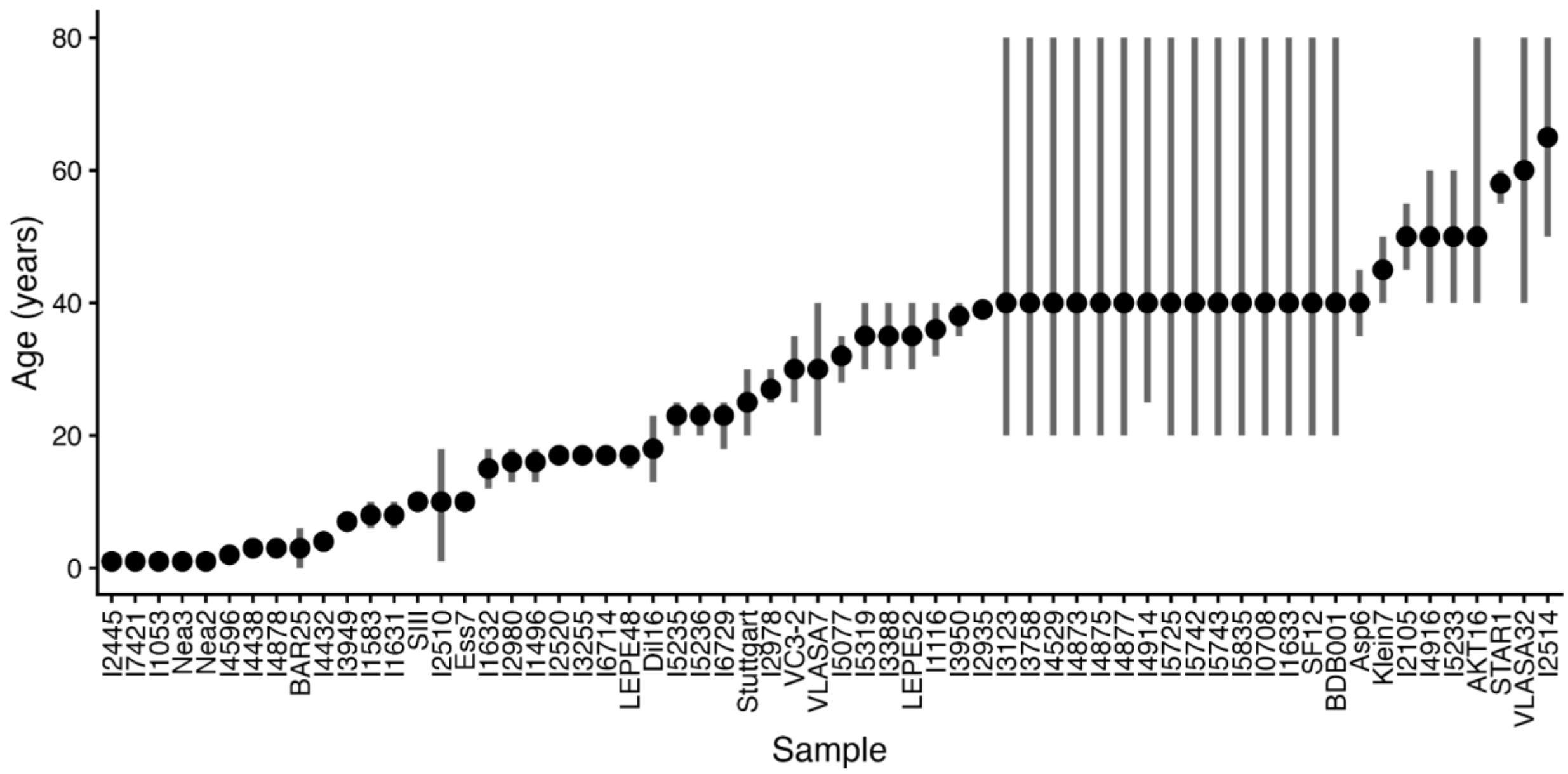

Supplementary Figure S2. Anthropological age-at-death estimates for the ancient individuals included in this study. Individuals are ordered by increasing mean estimated age-at-death. Points indicate the mean age estimate for each individual, and vertical lines represent the corresponding reported anthropological age range.

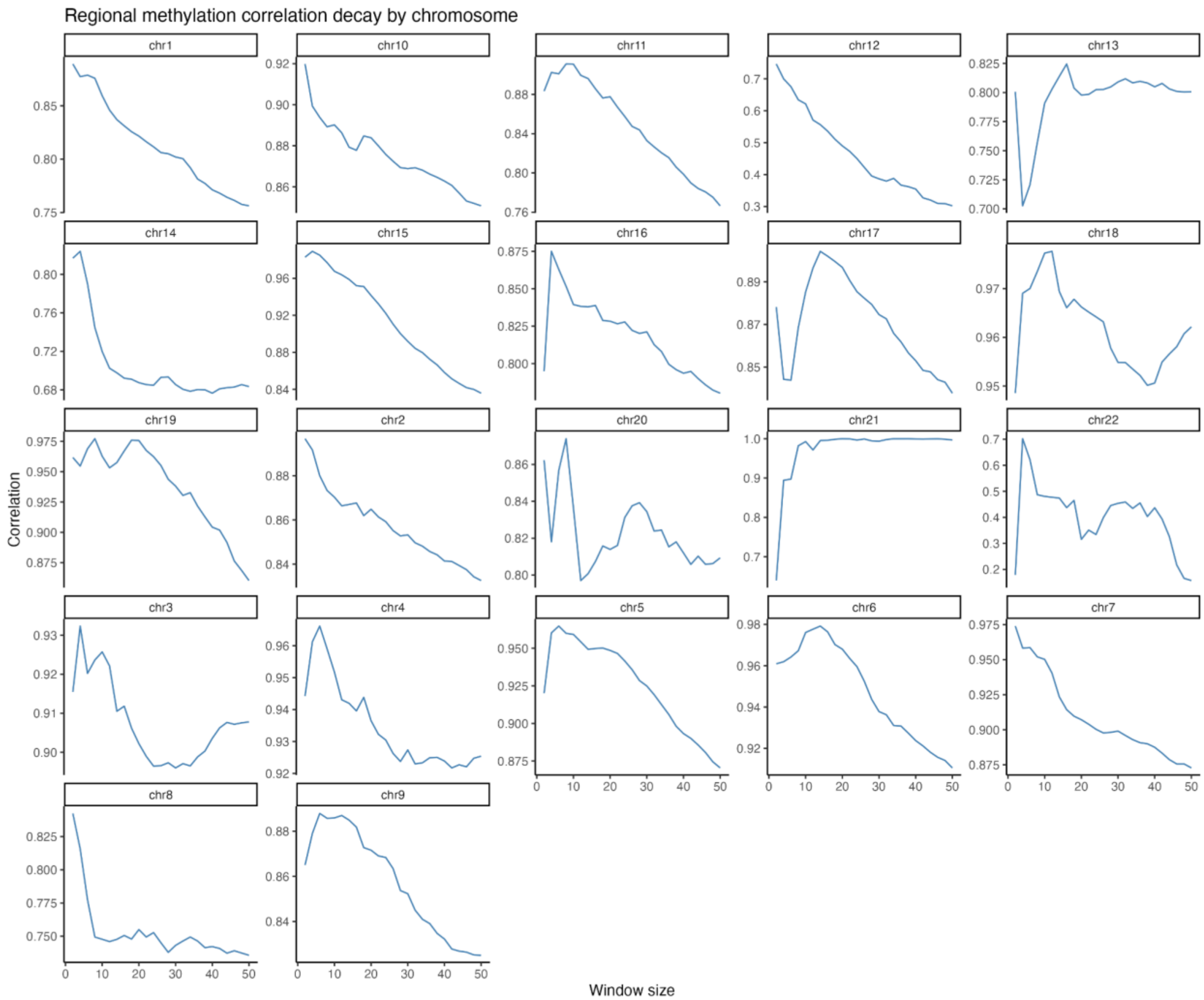

Supplementary Figure S3. Chromosome-wide decay of regional methylation correlation in a modern WGBS sample. Pearson correlation coefficients between methylation levels in adjacent CpG windows were calculated along each chromosome using increasing window sizes (2–50 CpGs).

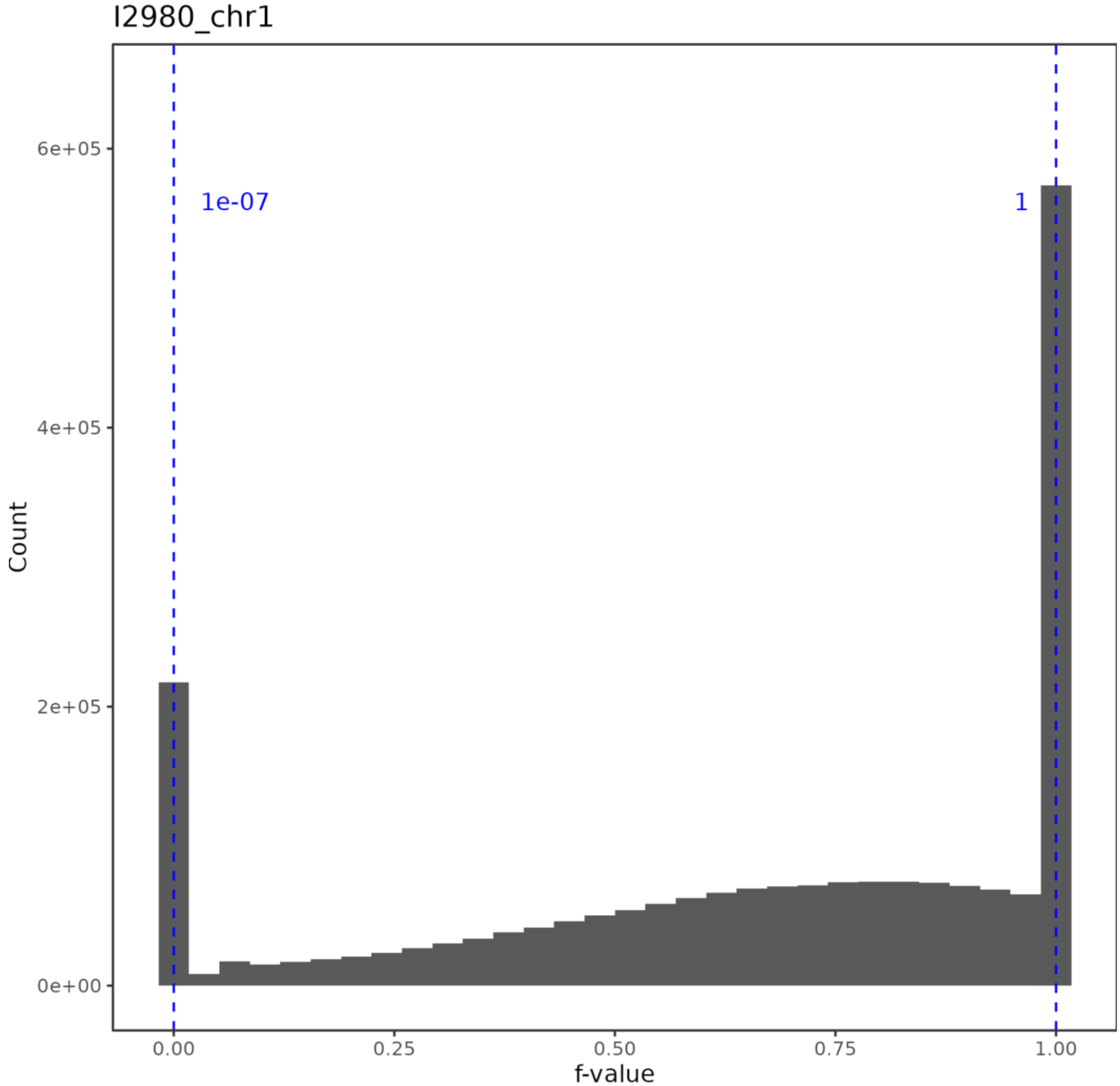

Supplementary Figure S4. Distribution of inferred methylation values across chromosome 1 in an ancient sample (I2980), for 10-CpG window. Histogram of raw inferred methylation values (F) across chromosome 1. X-axis shows F-values (0–1) and y-axis indicates the count of F-values. Blue dotted lines indicate the expected binomial peaks at values 0 and 1.

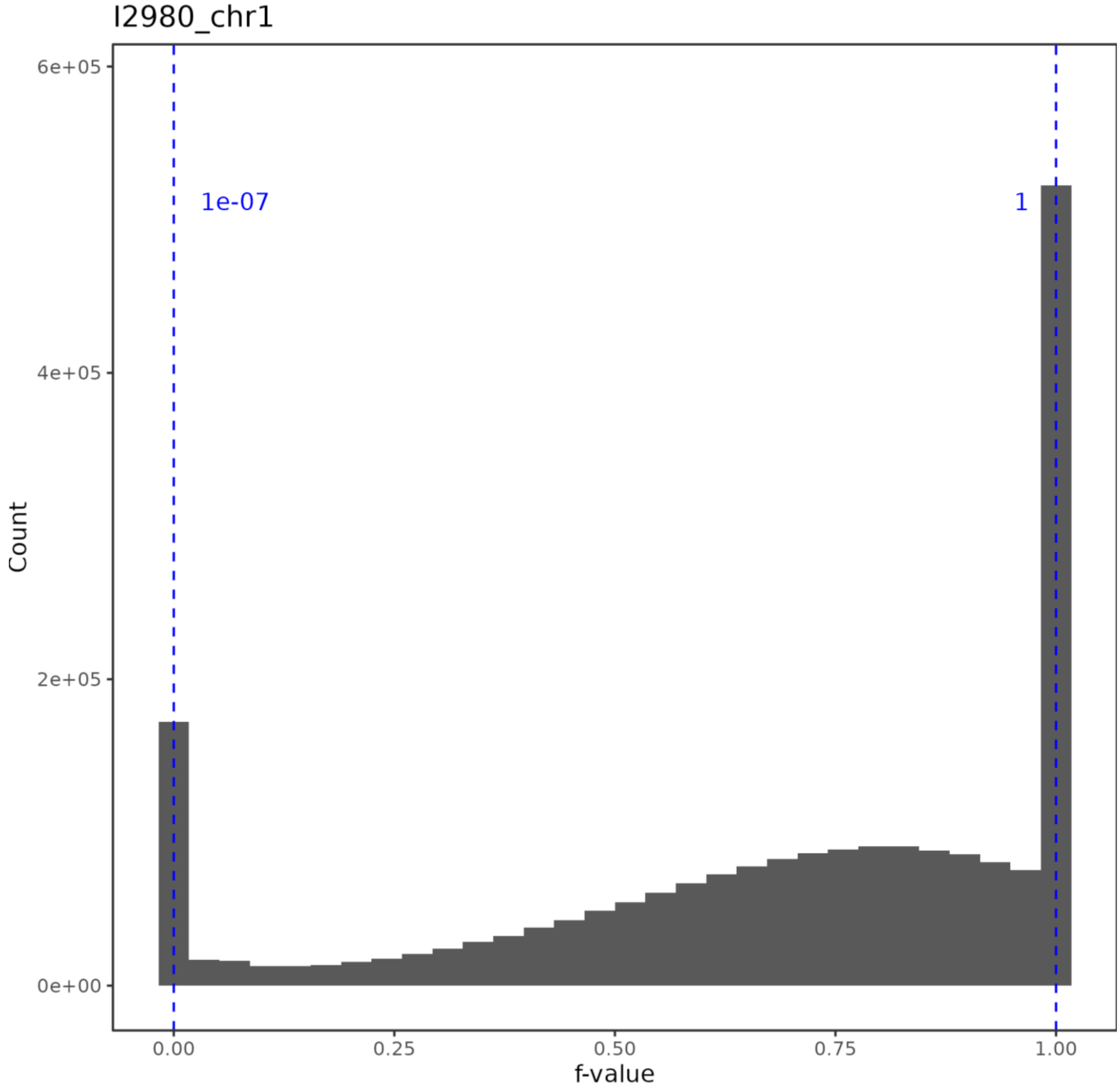

Supplementary Figure S5. Distribution of inferred methylation values across chromosome 1 in an ancient sample (I2980), for 15-CpG window. Histogram of raw inferred methylation values (F) across chromosome 1. X-axis shows F-values (0–1) and y-axis indicates the count of F-values. Blue dotted lines indicate the expected binomial peaks at values 0 and 1.

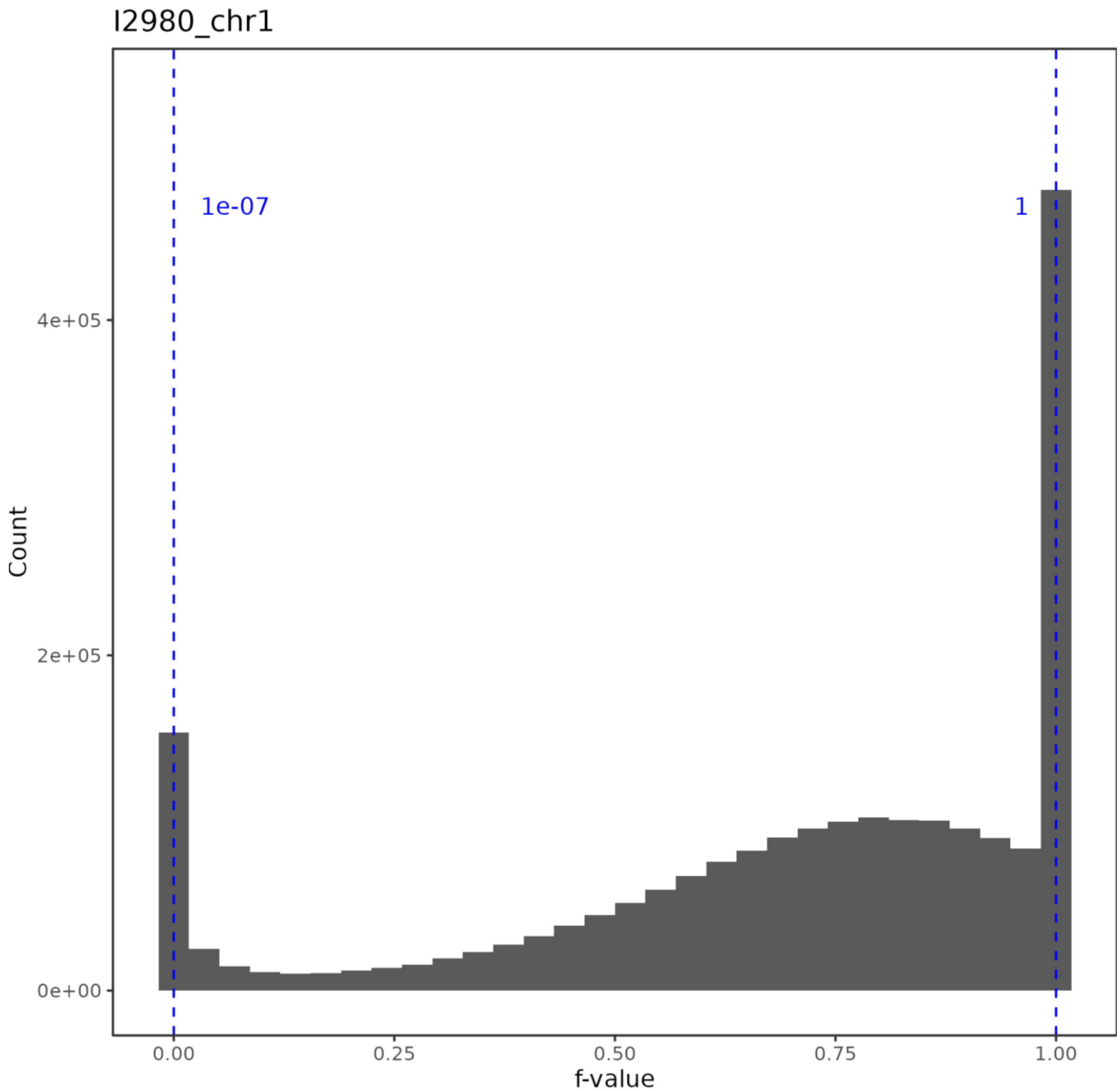

Supplementary Figure S6. Distribution of inferred methylation values across chromosome 1 in an ancient sample (I2980), for 20-CpG window. Histogram of raw inferred methylation values (F) across chromosome 1. X-axis shows F-values (0–1) and y-axis indicates the count of F-values. Blue dotted lines indicate the expected binomial peaks at values 0 and 1.

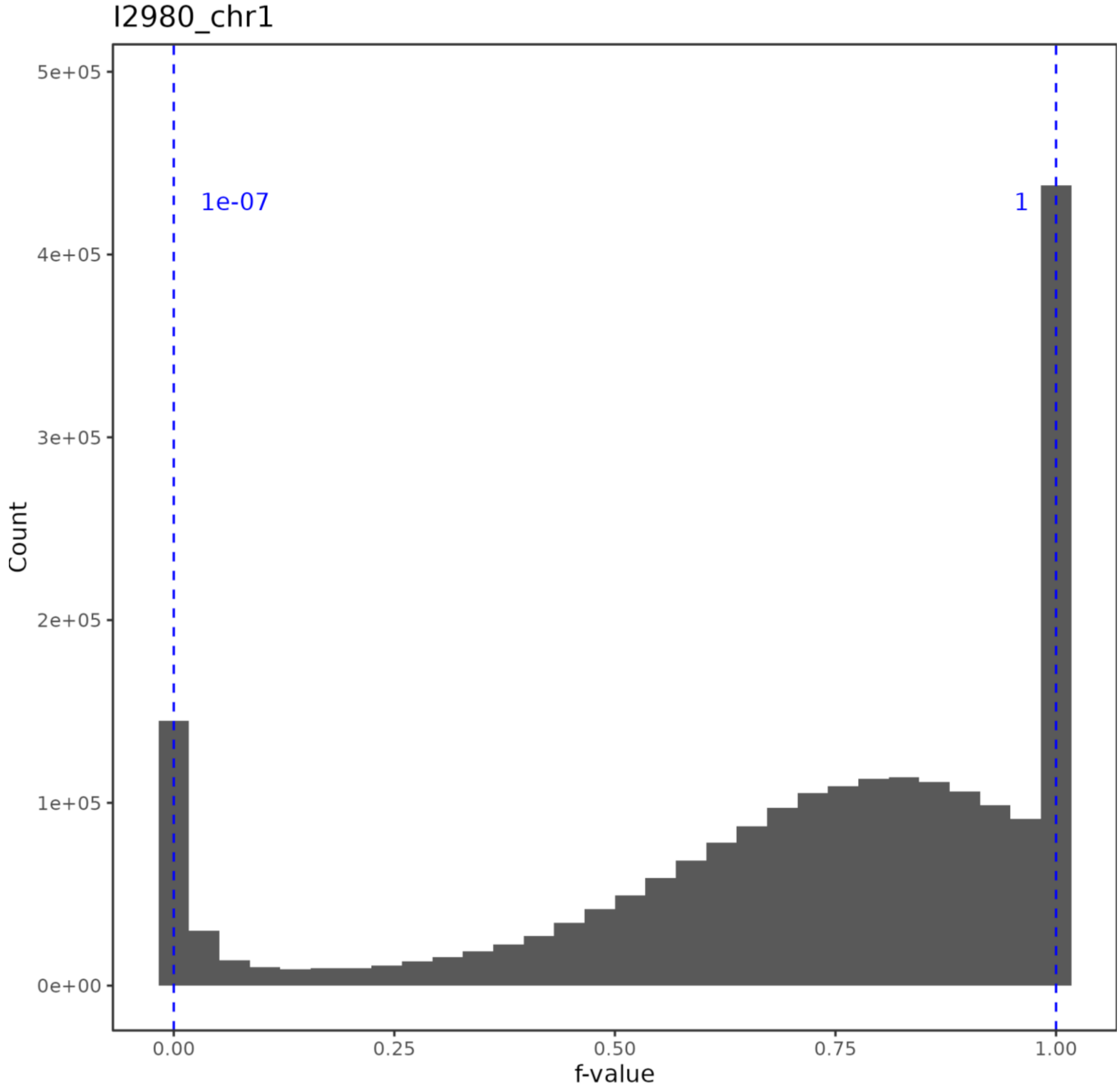

Supplementary Figure S7. Distribution of inferred methylation values across chromosome 1 in an ancient sample (I2980), for 25-CpG window. Histogram of raw inferred methylation values (F) across chromosome 1. X-axis shows F-values (0–1) and y-axis indicates the count of F-values. Blue dotted lines indicate the expected binomial peaks at values 0 and 1.

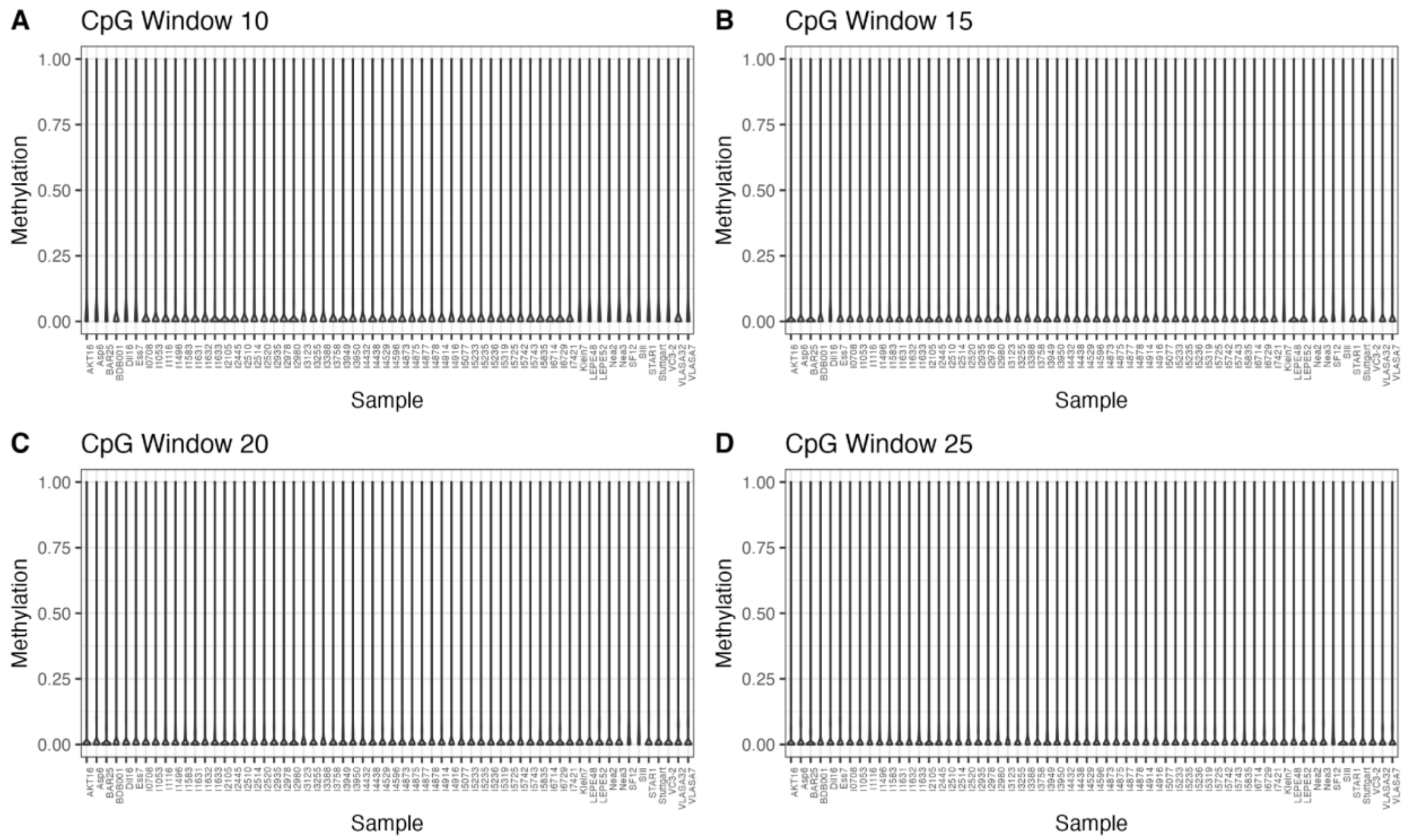

Supplementary Figure S8. CpG island methylation across ancient samples and different CpG windows. CpG island methylation values (y-axis) are shown for each sample (x-axis) across the four CpG window sizes (A–D).

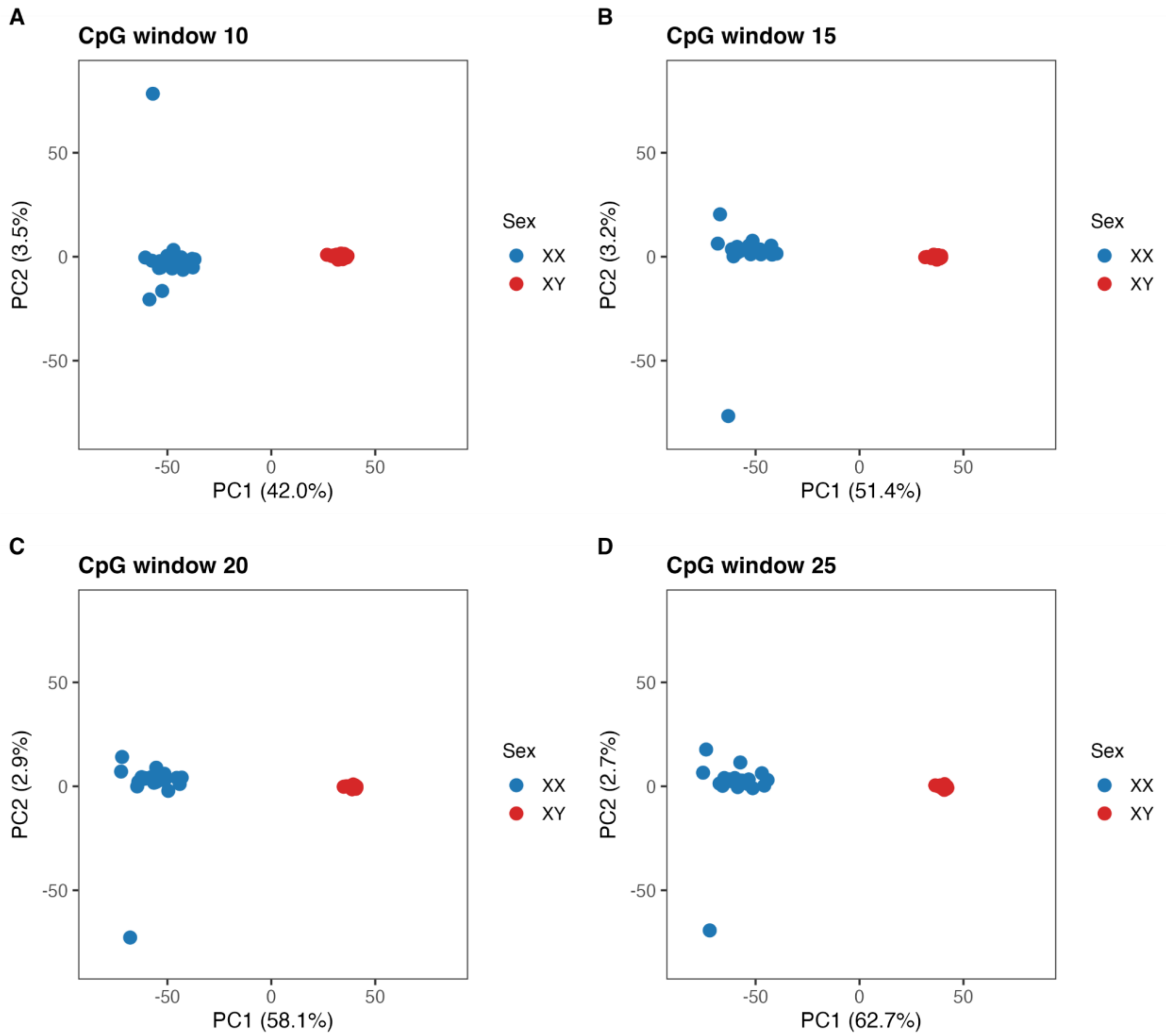

Supplementary Figure S9. Principal Component Analysis (PCA) of ~4,000 X-chromosome CpG sites after depth filtering, across different CpG sizes before correction. After retaining samples with mean depth ≥ 10X, females (blue) and males (red) separate clearly across the four CpG window sizes (panels A–D).

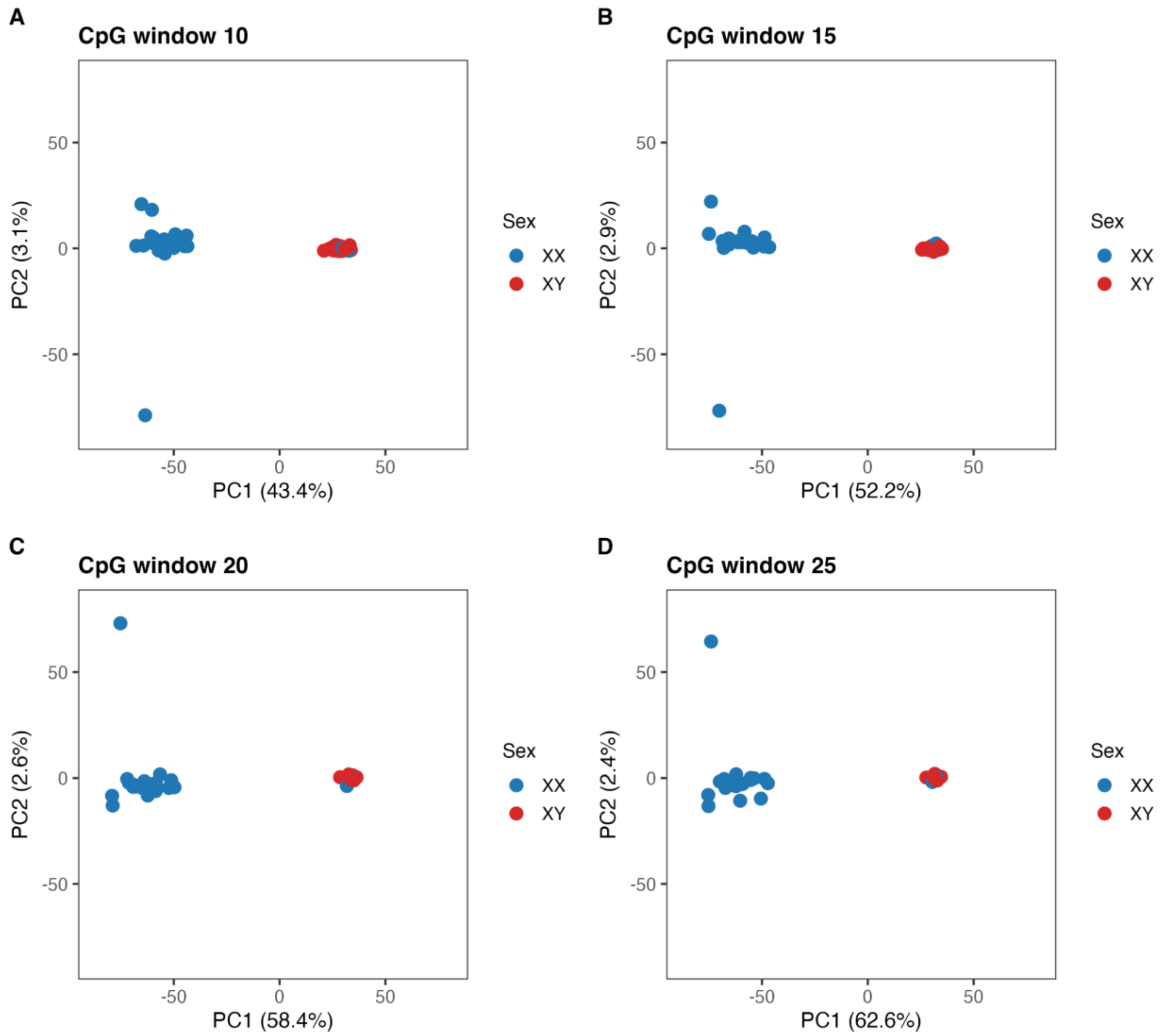

Supplementary Figure S10. Principal Component Analysis (PCA) of ~4,000 X-chromosome CpG sites before depth filtering, across different CpG sizes before correction. Two low-depth females (blue) cluster with males (red) Panels A–D represent the four CpG windows (10, 15, 20, 25).

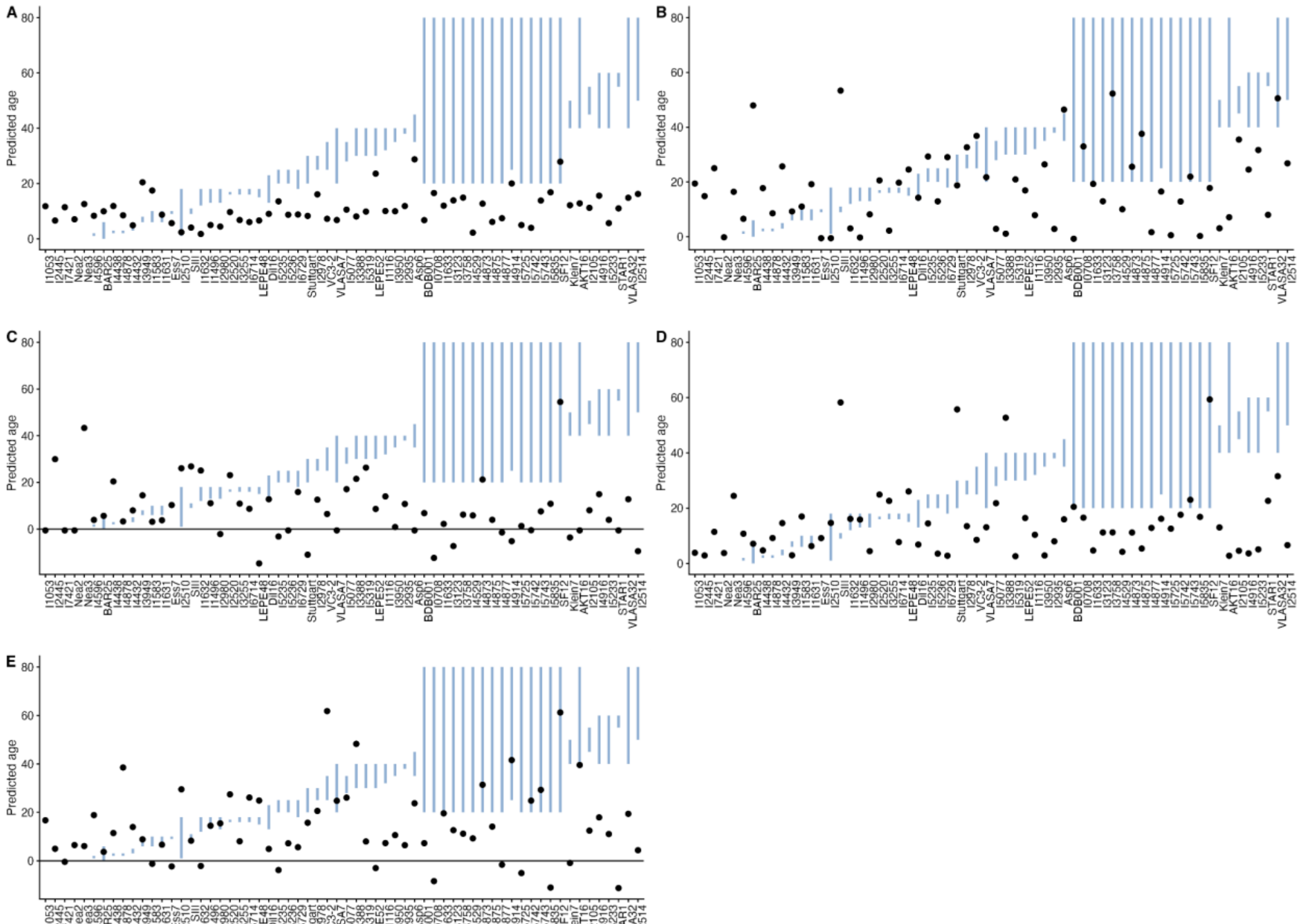

Supplementary Figure S11. Performance of epigenetic clocks on ancient methylomes before correction (CpG window 10). Panels show results for (A) UniversalPanMammalianClock1, (B) UniversalPanMammalianClock2, (C) BBT-APM, (D) the Horvath clock, and (E) 37-cpg. Vertical lines (blue) indicate anthropological age-at-death ranges, and points denote clock-derived predicted ages.

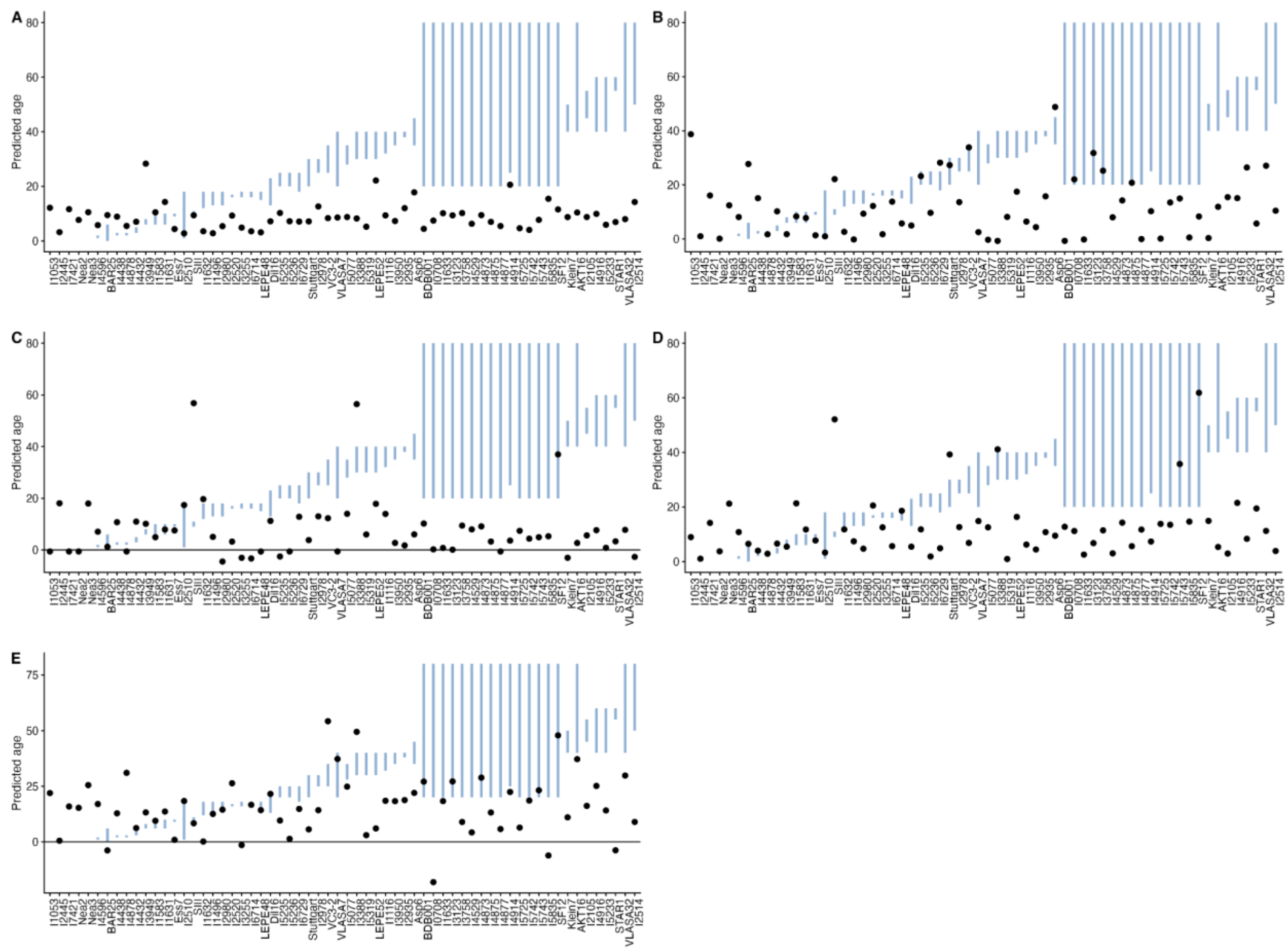

Supplementary Figure S12. Performance of epigenetic clocks on ancient methylomes before correction (CpG window 15). Panels show results for (A) UniversalPanMammalianClock1, (B) UniversalPanMammalianClock2, (C) BBT-APM, (D) the Horvath clock, and (E) 37-cpg. Vertical lines (blue) indicate anthropological age-at-death ranges, and points denote clock-derived predicted ages.

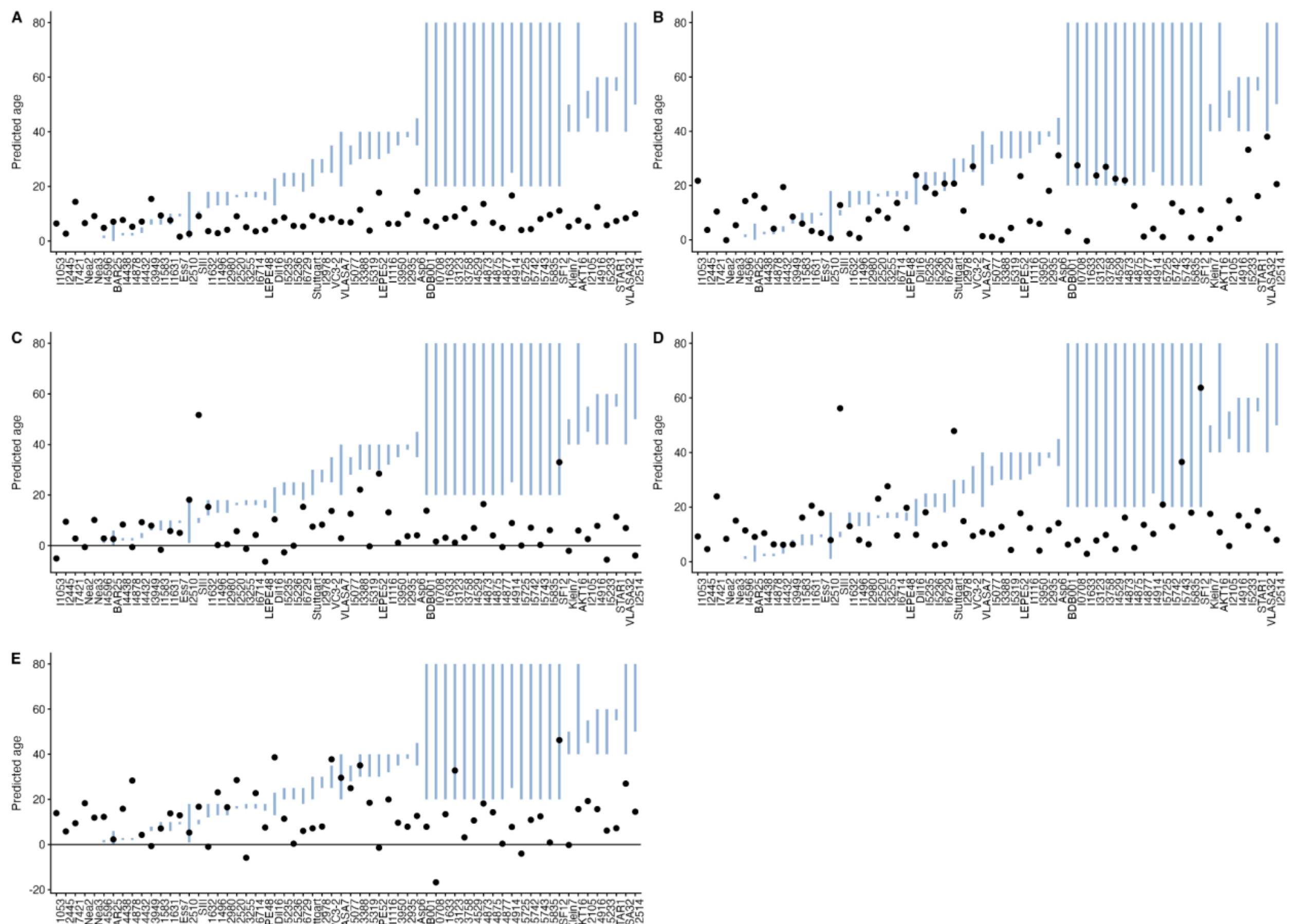

Supplementary Figure S13. Performance of epigenetic clocks on ancient methylomes before correction (CpG window 20). Panels show results for (A) UniversalPanMammalianClock1, (B) UniversalPanMammalianClock2, (C) BBT-APM, (D) the Horvath clock, and (E) 37-cpg. Vertical lines (blue) indicate anthropological age-at-death ranges, and points denote clock-derived predicted ages.

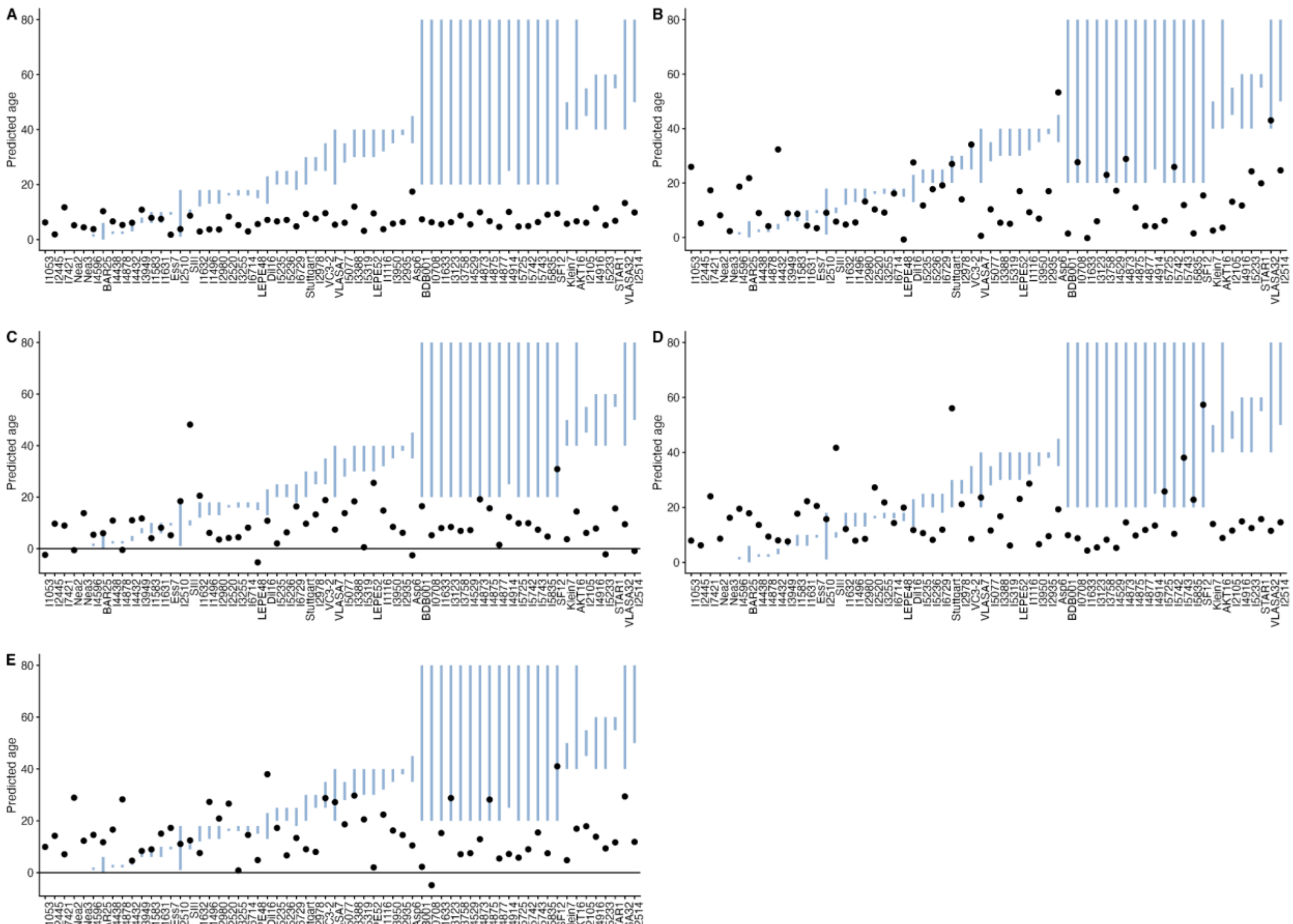

Supplementary Figure S14. Performance of epigenetic clocks on ancient methylomes before correction (CpG window 25). Panels show results for (A) UniversalPanMammalianClock1, (B) UniversalPanMammalianClock2, (C) BBT-APM, (D) the Horvath clock, and (E) 37-cpg. Vertical lines (blue) indicate anthropological age-at-death ranges, and points denote clock-derived predicted ages.

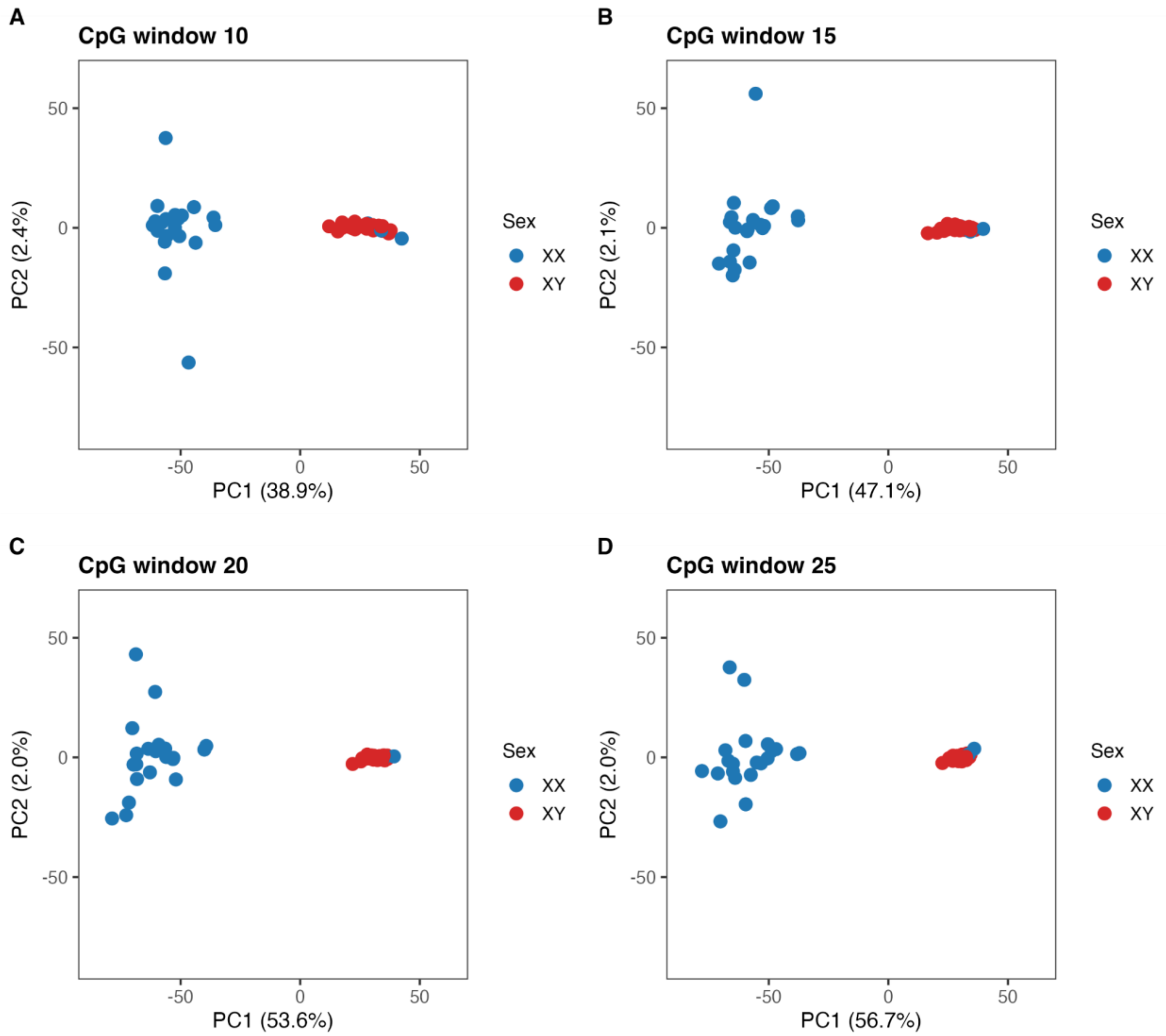

Supplementary Figure S15. Principal Component Analysis (PCA) of ~4,000 X-chromosome CpG sites before depth filtering, across different CpG sizes after correction. Two low-depth females (blue) cluster with males (red) Panels A–D represent the four CpG windows (10, 15, 20, 25).

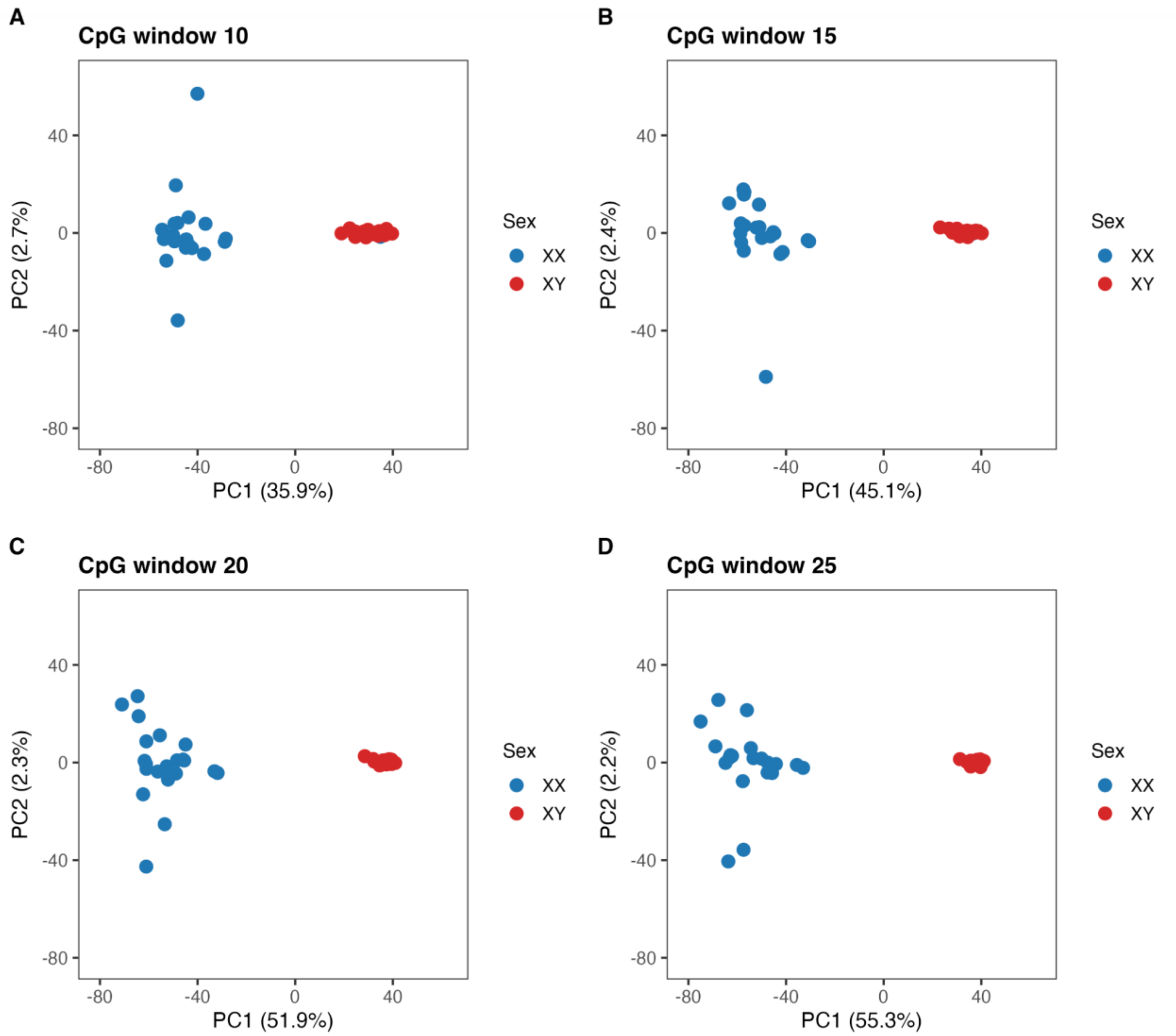

Supplementary Figure S16. Principal Component Analysis (PCA) of ~4,000 X-chromosome CpG sites after depth filtering, across different CpG sizes after correction. After retaining samples with mean depth ≥ 10X, females (blue) and males (red) separate clearly across the four CpG window sizes (panels A–D).

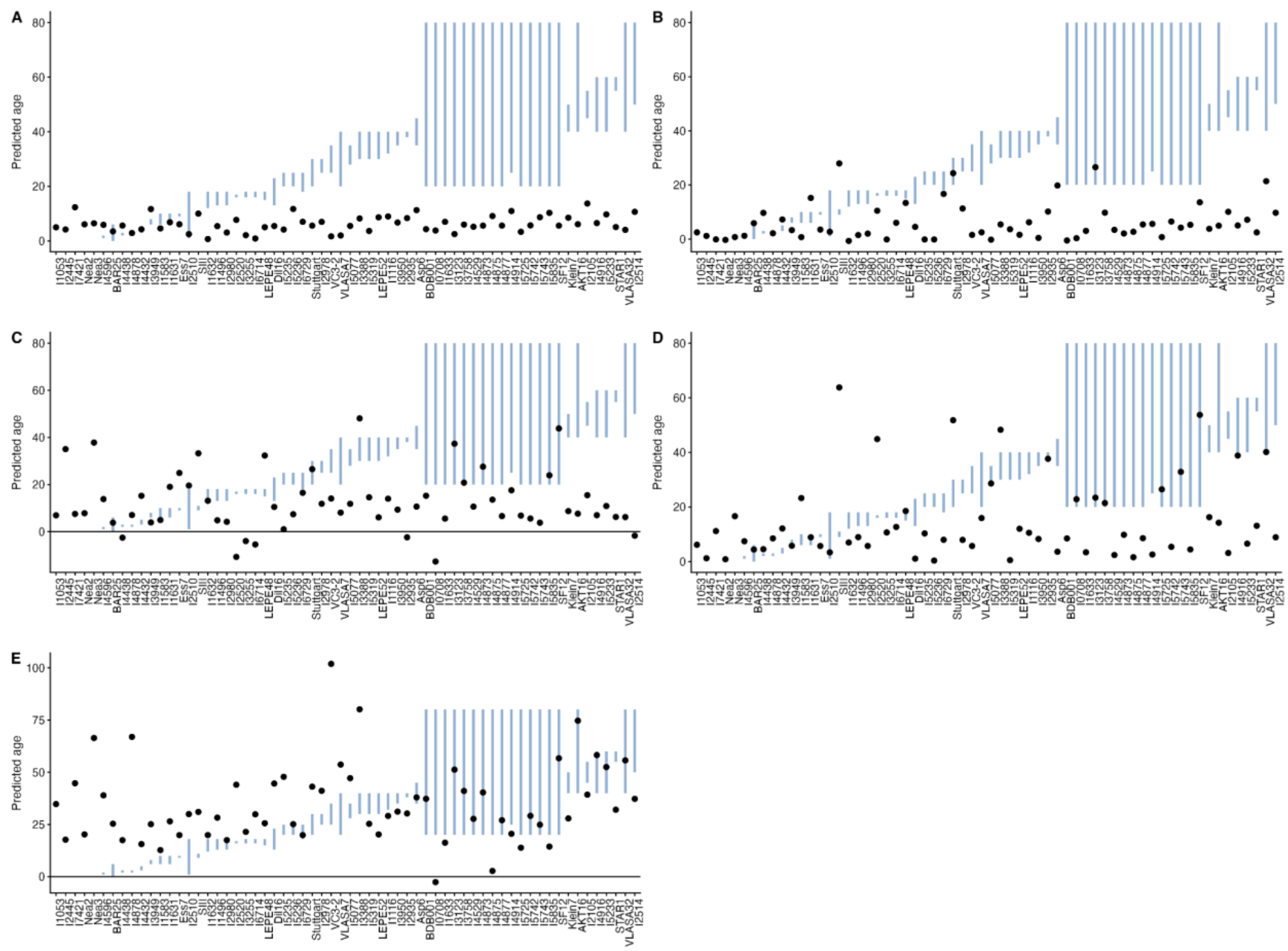

Supplementary Figure S17. Performance of epigenetic clocks on ancient methylomes after correction (CpG window 15). Panels show results for (A) UniversalPanMammalianClock1, (B) UniversalPanMammalianClock2, (C) BBT-APM, (D) the Horvath clock, and (E) the 37-CpG model. Vertical lines (blue) indicate anthropological age-at-death ranges, and points denote clock-derived predicted ages.

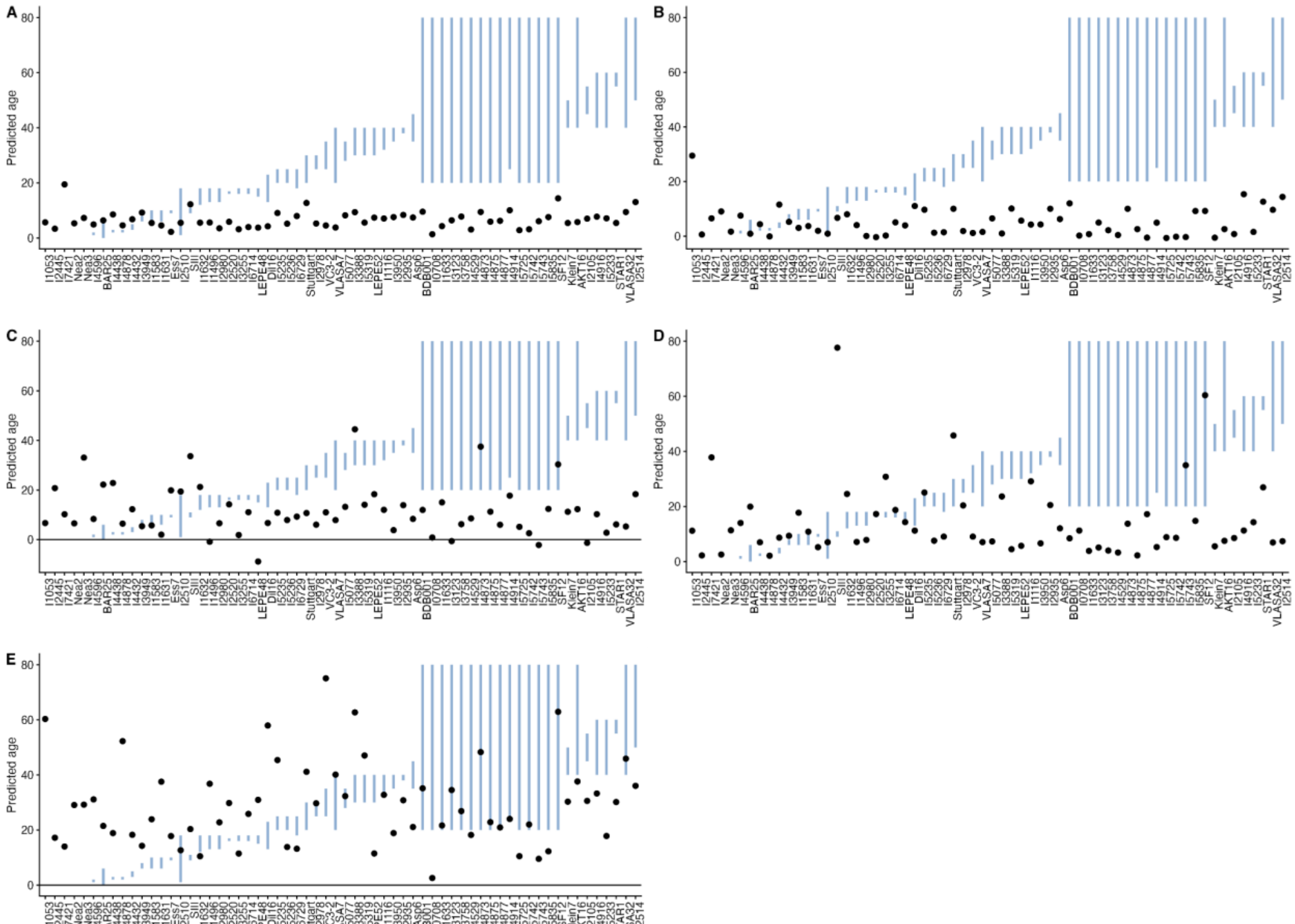

Supplementary Figure S18. Performance of epigenetic clocks on ancient methylomes after correction (CpG window 20). Panels show results for (A) UniversalPanMammalianClock1, (B) UniversalPanMammalianClock2, (C) BBT-APM, (D) the Horvath clock, and (E) the 37-CpG model. Vertical lines (blue) indicate anthropological age-at-death ranges, and points denote clock-derived predicted ages.

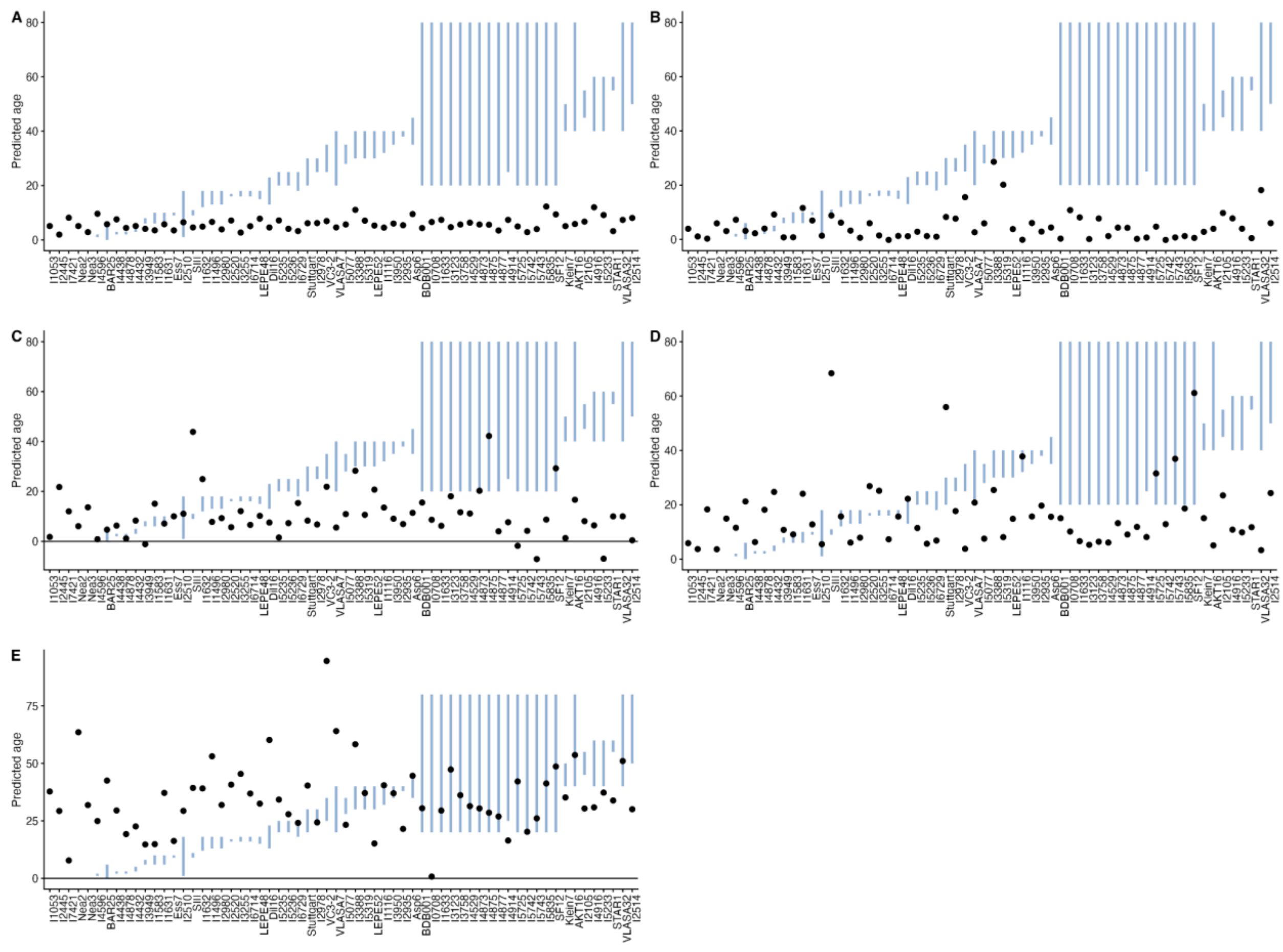

Supplementary Figure S19. Performance of epigenetic clocks on ancient methylomes after correction (CpG window 25). Panels show results for (A) UniversalPanMammalianClock1, (B) UniversalPanMammalianClock2, (C) BBT-APM, (D) the Horvath clock, and (E) the 37-CpG model. Vertical lines (blue) indicate anthropological age-at-death ranges, and points denote clock-derived predicted ages.

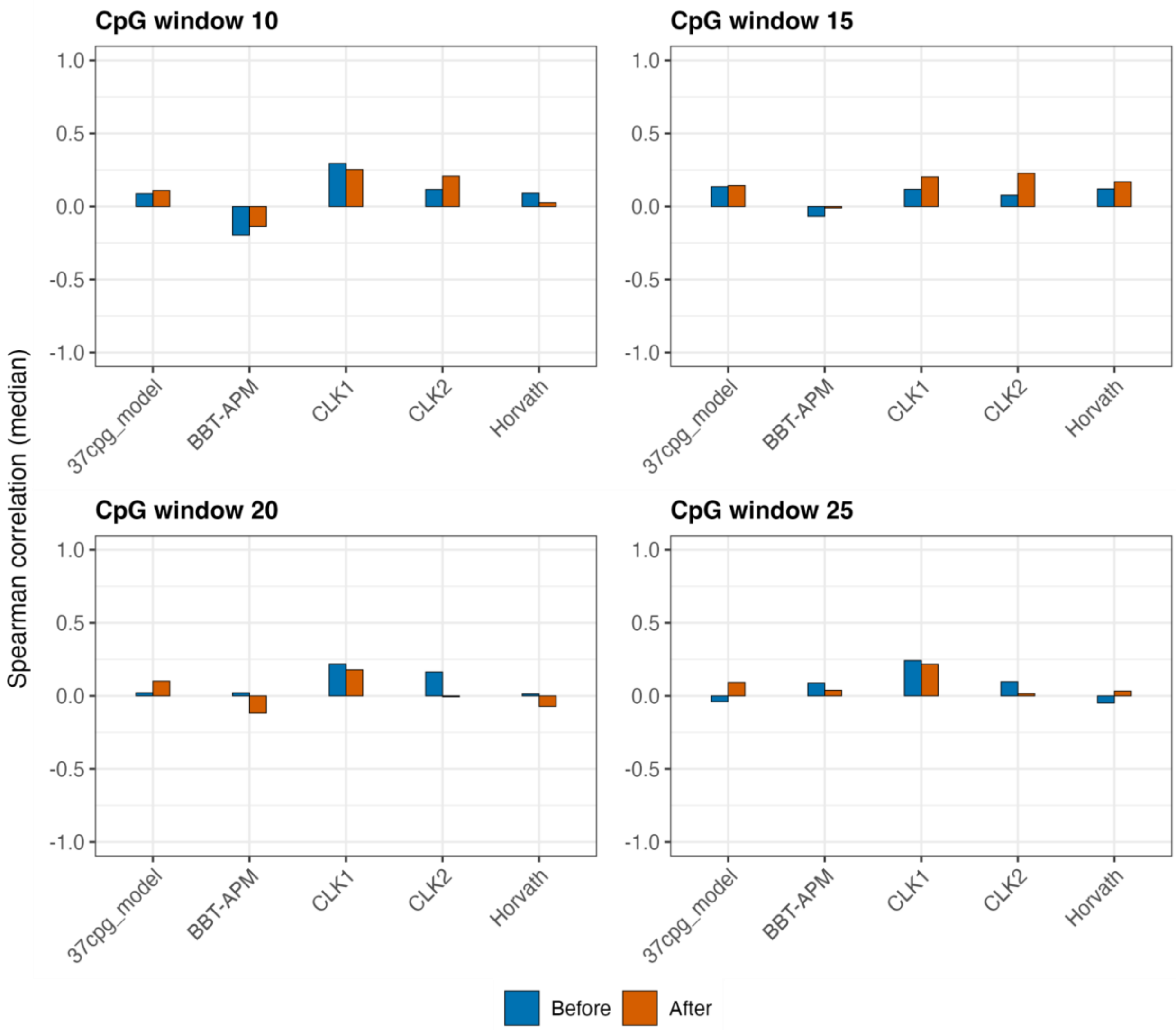

Supplementary Figure S20. Median Monte Carlo Spearman correlation before and after correction across modern epigenetic clocks and different CpG windows. For each CpG window size, bars show the median Monte Carlo Spearman correlation coefficient for the four modern epigenetic clocks, evaluated before (blue) and after (red) correction.

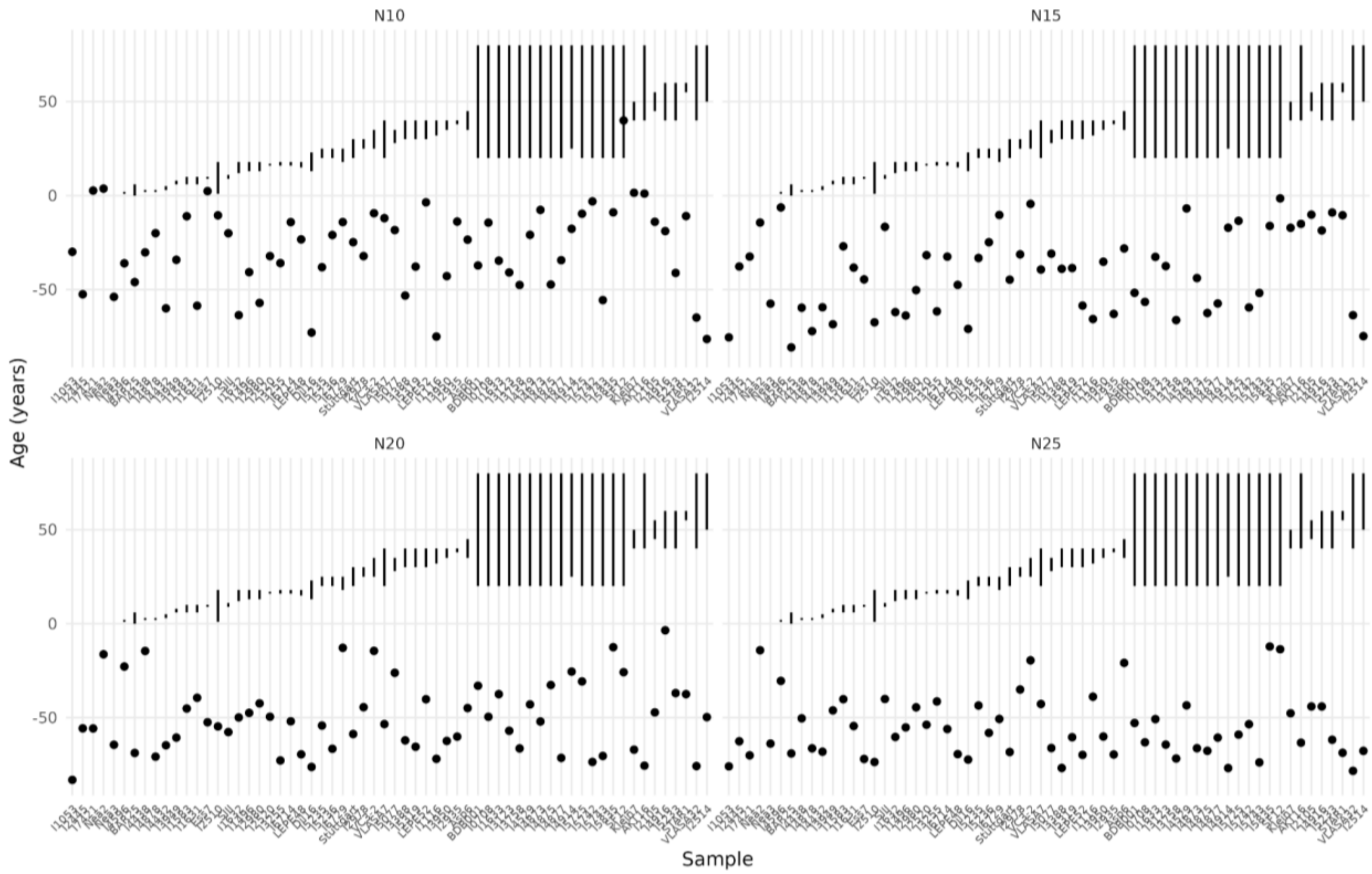

Supplementary Figure S21.Elastic net age predictions of ancient samples, trained on modern data, across the four CpG windows. Predicted ages (y-axis) are shown for each sample (x-axis) across the four CpG windows (N10–N25). Each point represents one individual and vertical lines indicate anthropological age-at-death ranges.

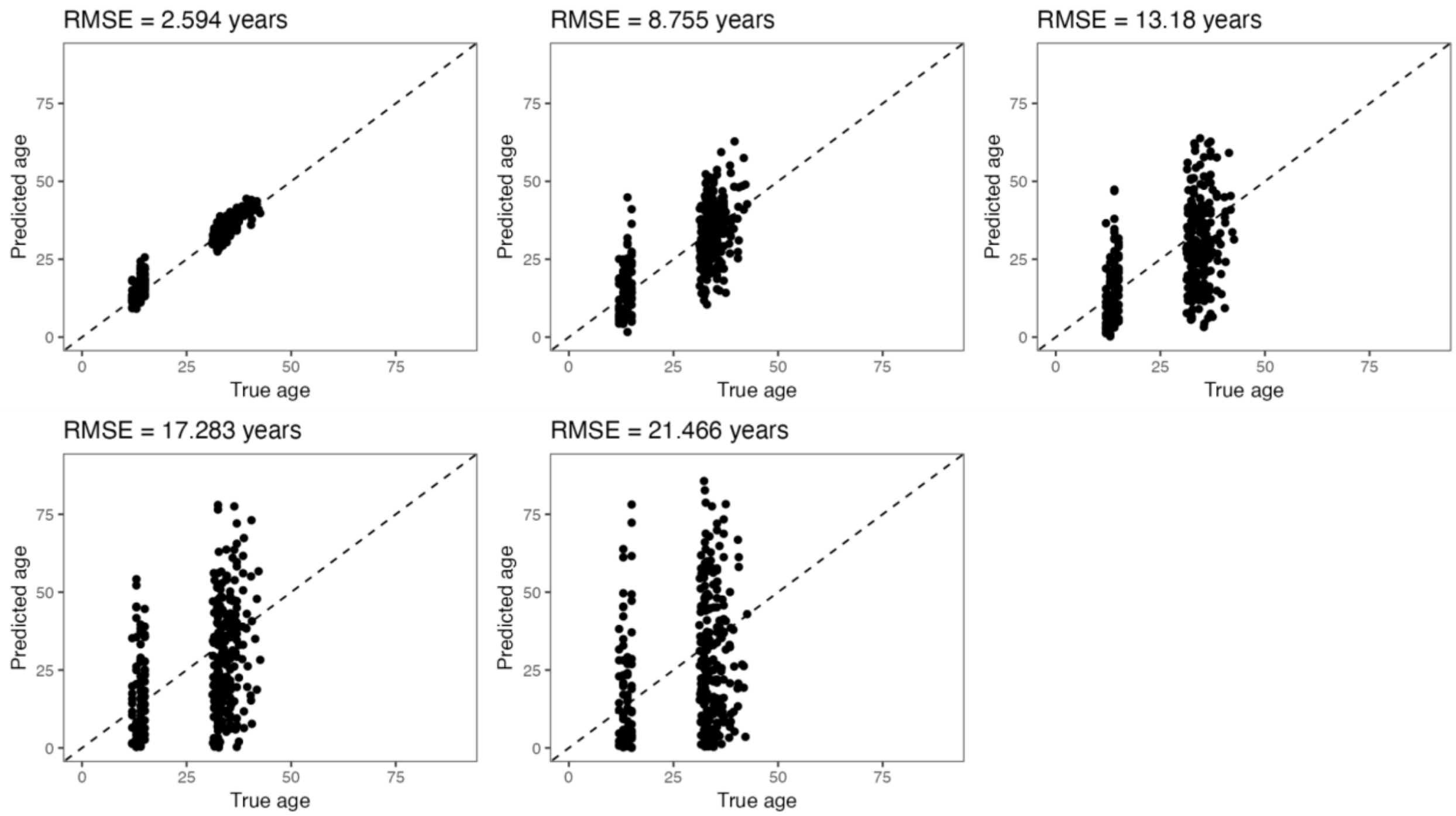

Supplementary Figure S22. Age prediction under increasing methylation perturbation. Predicted ages are shown across five conditions: unperturbed methylation values and four progressively increasing levels of added noise.

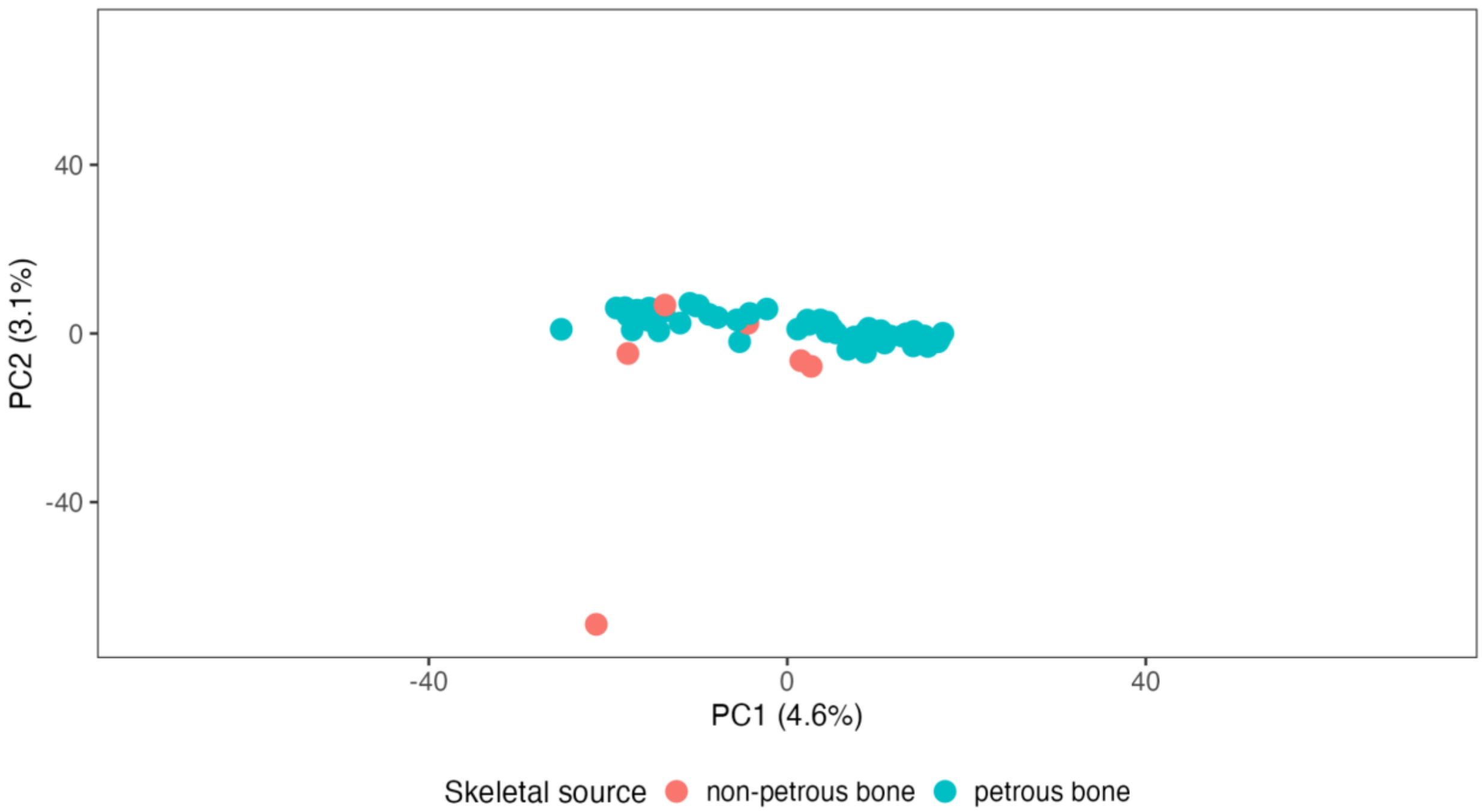

Supplementary Figure S23. Principal Component Analysis (PCA) of age-associated CpG sites after correction. Each point represents an ancient individual, colored by skeletal source (petrous vs non-petrous).
